# ‘Educated’ Osteoblasts Reduce Osteoclastogenesis in a Bone-Tumor Mimetic Microenvironment

**DOI:** 10.3390/cancers13020263

**Published:** 2021-01-12

**Authors:** Alexus D. Kolb, Jinlu Dai, Evan T. Keller, Karen M. Bussard

**Affiliations:** 1 Department of Cancer Biology, Sidney Kimmel Cancer Center, Thomas Jefferson University, Philadelphia, PA 19107, USA; axk182@students.jefferson.edu; 2 Department of Urology and Biointerfaces Institute, University of Michigan, Ann Arbor, MI 48109, USA; jldai@med.umich.edu (J.D.); etkeller@med.umich.edu (E.T.K.)

**Keywords:** osteoclast, osteoblast, breast cancer, metastasis, bone, osteoclastogenesis, tumor microenvironment

## Abstract

**Simple Summary:**

Patients with late-stage bone metastatic breast cancer experience skeletal related events, including osteolytic lesion formation, due to overactive osteoclast bone resorption. It is well-known that osteoclast function is altered by breast cancer cells in bone. Breast cancer cells stimulate osteoblasts to secrete factors that initiate osteoclast differentiation and activation. Our lab has previously identified a novel subpopulation of osteoblasts in the bone-tumor microenvironment called “educated” osteoblasts (EOs) that alter breast cancer cell proliferation. The aim of this study was to identify how osteoclasts are affected by EOs during metastatic breast cancer progression in bone. Our results demonstrated that pre-osteoclast interaction with EOs reduces osteoclast formation and bone resorption in a bone-tumor mimetic microenvironment. Furthermore, we identified that altered osteoclast formation can be modulated, in part, by tumor necrosis factor alpha (TNFα). Overall, our data demonstrate osteoclastogenesis is reduced by EO cells, suggesting EO cells have a protective effect in bone and exert an inhibitory effect on tumor progression.

**Abstract:**

Breast cancer (BC) metastases to bone disrupt the balance between osteoblasts and osteoclasts, leading to excessive bone resorption. We identified a novel subpopulation of osteoblasts with tumor-inhibitory properties, called educated osteoblasts (EOs). Here we sought to examine the effect of EOs on osteoclastogenesis during tumor progression. We hypothesized that EOs affect osteoclast development in the bone-tumor niche, leading to suppressed pre-osteoclast fusion and bone resorption. Conditioned media (CM) was analyzed for protein expression of osteoclast factors receptor activator of nuclear factor kappa-β ligand (RANKL), osteoprotegerin (OPG), and tumor necrosis factor alpha (TNFα) via ELISA. EOs were co-cultured with pre-osteoclasts on a bone mimetic matrix to assess osteoclast resorption. Pre-osteoclasts were tri-cultured with EOs plus metastatic BC cells and assessed for tartrate-resistance acid phosphatase (TRAP)-positive, multinucleated (≥3 nuclei), mature osteoclasts. Tumor-bearing murine tibias were stained for TRAP to determine osteoclast number in-vivo. EO CM expressed reduced amounts of soluble TNFα and OPG compared to naïve osteoblast CM. Osteoclasts formed in the presence of EOs were smaller and less in number. Upon co-culture on a mimetic bone matrix, a 50% reduction in the number of TRAP-positive osteoclasts formed in the presence of EOs was observed. The tibia of mice inoculated with BC cells had less osteoclasts per bone surface in bones with increased numbers of EO cells. These data suggest EOs reduce osteoclastogenesis and bone resorption. The data imply EOs provide a protective effect against bone resorption in bone metastatic BC.

## 1. Introduction

Breast cancer is the second leading cause of cancer deaths in women in the United States [1]. Unfortunately, three-fourths of women diagnosed with primary breast cancer will experience metastatic disease [2]. Bone is a preferential site of breast cancer metastases [3,4]. Patients with bone metastases have a five-year survival rate of <10% [5]. Bone metastatic breast cancer patients present with lesions that are either osteoblastic, osteolytic, or a mix of osteoblastic and osteolytic [6]. Breast cancer bone metastatic lesions are most commonly osteolytic, in which patients experience complications including severe bone pain, fractures, and hypercalcemia due to excess bone resorption by osteoclasts [7,8].

Under normal conditions, bone remodeling is regulated by both bone-forming osteoblasts and bone-resorbing osteoclasts, with no net bone loss or gain [9,10]. However, when metastatic breast cancer cells invade the skeleton, bone remodeling is disrupted [11], whereby osteoclasts become overactive and resorb bone quicker than osteoblasts deposit new bone [12]. Due to excess bone resorption, bone metastases are associated with high morbidity and poor clinical outcome [13]. Patients with osteolytic bone metastatic breast cancer are treated with bisphosphonates, which are aimed at impairing the activity of bone-resorbing osteoclasts [14,15]. However, bisphosphonates are not curative for the lesions already present, and they do not stimulate new bone deposition by osteoblasts [6]. Currently, there are a few therapies available to stimulate osteoblast activity; however, each has its limitations. Romosozumab is a drug that was recently approved by the FDA for treatment of osteoporosis and functions by inhibiting expression of sclerostin, a glycoprotein and potent inhibitor of bone deposition [16,17]. However, a global phase III active-controlled fracture study in postmenopausal women with osteoporosis at high risk (ARCH) study revealed Romosozumab caused a 31% increase in major adverse cardiovascular events in patients taking the drug when compared to alendronate, the current standard of care [18]. Furthermore, teriparatide (sold under the brand name Forteo^®^) is a synthetic form of human parathyroid hormone that acts to promote bone density. However, some studies have suggested that teriparatide promotes osteosarcoma in rat models and may drive tumor progression in pre-malignant lesions in humans [19]. 

**Figure 1:**
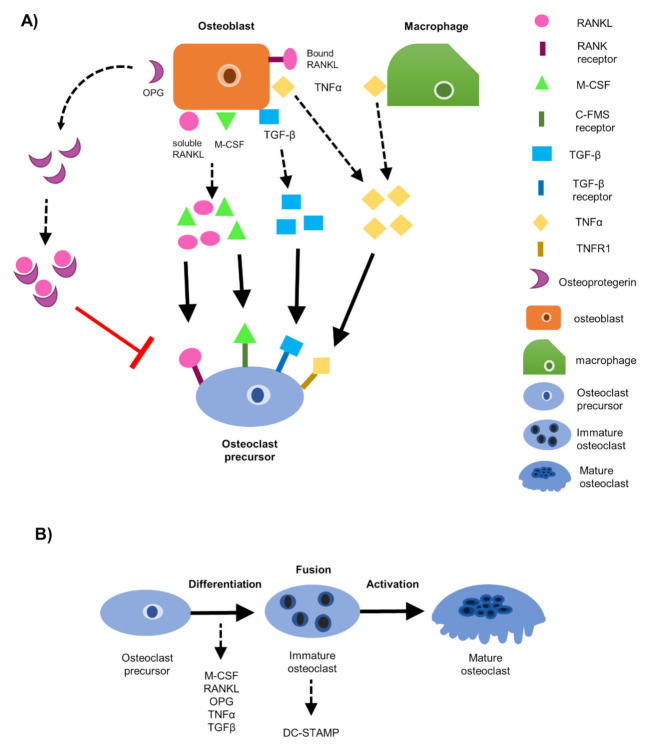
Osteoblasts are important regulators of osteoclast differentiation. (**A**) During bone remodeling, osteoblasts recruit osteoclast progenitors to bone, whereby they secrete factors needed for osteoclast differentiation. Osteoblasts secrete macrophage-colony factor (M-CSF) and receptor activator of nuclear-factor kappa-β ligand (RANKL). The binding of M-CSF to the colony-stimulating factor-1 (C-FMS) receptor commits osteoclast progenitor cells to osteoclast linage and initiates osteoclast differentiation. RANKL is also an important molecule for osteoclast differentiation. RANKL can exist in a bound form on osteoblasts or a soluble form secreted from osteoblasts. In this case, soluble RANKL is secreted from osteoblasts and binds the receptor activator of nuclear factor kappa-β (RANK) receptor on osteoclast progenitors, further initiating differentiation of pre-osteoclasts. Additionally, osteoclastogenesis can be initiated by RANKL-independent mechanisms, including transforming growth factor-beta (TGF-β) and tumor necrosis factor-alpha (TNFα). TGF-β, secreted from osteoblasts, binds to the TNF-β receptor complex on osteoclast progenitor cells to independently initiate osteoclast differentiation. TNFα, secreted from osteoblasts or macrophages, binds to the tumor necrosis factor receptor 1 (TNFR1) on osteoclast progenitor cells and can also independently initiate osteoclast differentiation. To combat excess bone degradation, osteoblasts secrete osteoprotegerin (OPG), a decoy receptor for RANKL. OPG acts as a decoy receptor, where it can bind soluble RANKL. In turn, soluble RANKL is inhibited from binding to the RANK receptor on osteoclast progenitors. In this way, osteoclastogenesis in inhibited. (**B**) Osteoclast differentiation is regulated by the expression of M-CSF, RANKL, OPG, TNFα, and TGF-β. Once osteoclast differentiation is initiated, osteoclast progenitors fuse together with the help of the key osteoclast fusion regulator dendrocyte expressed seven transmembrane protein (DC-STAMP) to become immature osteoclasts. Upon activation, immature osteoclasts become mature osteoclasts capable of resorbing bone.

It was previously thought that bone metastatic breast cancer cells directly mediate bone destruction [7], but most evidence indicates that metastatic breast cancer cells work in a paracrine manner to initiate bone destruction by osteoclasts [20,21]. In order to understand bone destruction by osteoclasts during breast cancer metastasis, it is first crucial to understand how osteoblasts and osteoclasts interact during normal bone remodeling. During normal bone remodeling, osteoblasts recruit osteoclast progenitors to the site of bone remodeling and secrete macrophage colony stimulating factor (M-CSF) and receptor activator of nuclear factor kappa-β ligand (RANKL) to initiate osteoclast differentiation [10]. RANKL can exist in two forms on osteoblasts, a bound form and a soluble form, both of which have been shown to initiate osteoclast differentiation [22] (Figure 1A). M-CSF and RANKL, both secreted from osteoblasts, bind to the colony-stimulating factor-1 (C-FMS) receptor and the receptor activator of nuclear factor kappa-β (RANK) receptor, respectively, on osteoclast progenitor cells [23] (Figure 1A). Osteoblasts also secrete osteoprotegerin (OPG), a decoy receptor for soluble RANKL [24] (Figure 1A). OPG binds soluble RANKL and inhibits it from binding to the RANK receptor on osteoclast progenitors [25] (Figure 1A). In this way, osteoclastogenesis is inhibited, and bone homeostasis is maintained [26].

Prior studies have also identified RANKL-independent mechanisms capable of inducing osteoclast differentiation [27]. For example, during normal bone remodeling, transforming growth factor beta (TGF-β) produced by osteoblasts and tumor necrosis factor alpha (TNFα), produced by osteoblasts and macrophages, have been shown to induce osteoclast differentiation independently of RANKL [28,29] (Figure 1A). TGF-β was sufficient to induce formation of TRAP+ osteoclasts in cultures of human monocytes or RAW264.7 pre-osteoclast cells independent of RANKL, TNFα, IL-6, or IL-11 [29]. Alternatively, TNFα can bind to the tumor necrosis factor receptor 1 (TNFR1) and can stimulate downstream osteoclast differentiation signaling pathways [30]. Other studies have shown that TNFα can also work in a paracrine manner by stimulating RANKL secretion by osteoblasts [31].

Once osteoclast differentiation is initiated, dendrocyte expressed seven transmembrane protein (DC-STAMP), the master regulator of osteoclast fusion, is activated and osteoclast progenitors fuse together [32,33]. After fusion occurs, immature osteoclasts become large, multinucleated (≥3 nuclei) mature osteoclasts capable of resorbing bone [23,34] (Figure 1B).

Under pathological conditions, such as in bone metastatic breast cancer, bone remodeling is disrupted [11]. This complex interaction between osteoblasts, osteoclasts, and bone metastatic breast cancer cells has been described as “the vicious cycle” of bone degradation [3,35]. In this state, breast cancer cells secrete factors, such as parathyroid hormone related protein (PTHrP) or TNFα, that increase the secretion of soluble RANKL from osteoblasts [35]. Excess soluble RANKL binds to the RANK receptor on osteoclast progenitor cells, in turn, activating osteoclastogenesis and increasing bone resorption [35]. Importantly, OPG secretion from osteoblasts is inhibited, which increases RANKL concentrations, further driving bone destruction [36]. Sustained bone degradation by osteoclasts increases the release of sequestered cytokines, growth factors, and minerals in the bone matrix, which breast cancer cells use to fuel this “vicious” cycle [35,37]. Consequently, osteoclasts are constitutively overactive, resorbing bone at a faster rate than osteoblasts deposit new bone, resulting in overall net bone loss with no new bone deposition, and in turn, promoting cancer growth [35,38].

It is well established that osteoblasts directed by metastatic breast cancer cells facilitate osteoclast differentiation and activation to promote cancer cell growth in late-stage disease [39]. Our laboratory and others have shown that osteoblasts and osteoclasts are also important modulators of metastatic cancer cell proliferation during early-stage disease [40,41]. Lawson and colleagues found that osteoblasts suppress proliferation of multiple myeloma cells that had metastasized to the bone during early-stage disease [42]. They found that multiple myeloma cells that engaged in crosstalk with osteoblasts entered into a “dormant” state in bone, and could be maintained there as long as the interaction with osteoblasts in the bone matrix was sustained [42]. Upon interaction with activated osteoclasts, as opposed to osteoblasts, the authors found that the “dormant” multiple myeloma cells were re-awakened, and consequently contributed to tumor progression in the bone [42]. From these data, Lawson et al. concluded that interactions with osteoblasts in the bone niche suppress cancer cell proliferation (acting as an “off” switch to multiple myeloma cell proliferation), whereas interactions with osteoclasts in the bone niche promote cancer cell growth and progression (acting as an “on” switch) [42]. These data suggest that activated osteoclasts are important mediators in reactivation of dormant breast cancer cells.

Our laboratory recently identified two subpopulations of osteoblasts in the bone-tumor microenvironment. These two cell populations consist of “educated” osteoblasts, or “EO” cells, (defined by their high expression of RUNX2, OPN, and OCN, but low expression of IL-6 and alpha-SMA, among others) and naïve, or “uneducated”, osteoblasts (defined by their high expression of RUNX2, OPN, OCN, IL-6, and alpha-SMA, among others) [39,40]. Naïve osteoblasts differ from “EO” cells in protein marker expression, effect on breast cancer cell proliferation, as well as contribution to tumor progression in the bone-tumor niche [39,40]. Importantly, our prior data indicate that EO cells have a tumor-inhibitory effect in the bone-tumor microenvironment.

Because EO cells exhibit altered properties compared to naïve osteoblasts, we wanted to determine how osteoclastogenesis was affected by EO cells. We hypothesized that EOs reduce osteoclastogenesis and subsequent bone resorption. In the present study, we found that soluble factors from EO conditioned media or direct contact with EO cells produced osteoclasts that were less in number and smaller in size. We further demonstrated that osteoclasts produced in the presence of EO cells had reduced resorptive activity compared to osteoclasts produced in the presence of naïve osteoblasts. We also observed decreased DC-STAMP protein expression in osteoclasts exposed to EO conditioned media compared to osteoclasts exposed to naïve osteoblast conditioned media. To understand how osteoclastogenesis is impacted by breast cancer cells, we tri-cultured pre-osteoclasts with breast cancer cells plus EO cells or naïve osteoblasts and analyzed osteoclast formation. We found that osteoclasts produced in the presence of breast cancer cells plus EO cells were less in number compared to osteoclasts produced in the presence of breast cancer cells plus naïve osteoblasts. To recapitulate our in-vitro results in-vivo, we used an intratibial model of bone metastasis and showed that the presence of EO cells decreased osteoclast formation in-vivo.

To determine the mechanism for decreased osteoclastogenesis, we analyzed conditioned media from EO cells and naïve osteoblasts for alterations in soluble protein production of RANKL, OPG, and TNFα. We found that OPG and TNFα soluble protein production was significantly decreased in EO conditioned media compared to naïve osteoblast conditioned media. We demonstrated that EO-altered osteoclast formation could be modulated with recombinant TNFα protein and that neutralization of TNFα restored osteoclast formation to levels seen with the addition of EO conditioned media alone. Thus, our data suggest that EO cells alter osteoclast formation and resorption. In agreement with current knowledge, our data further suggest TNFα is a potent regulator of osteoclast formation.

## 2. Materials and Methods

### 2.1. Cells

All cells tested negative for *Mycoplasma spp.* infection using a MycoSensor PCR Assay kit (Agilent Technologies, Santa Clara, CA, USA). All cells were cultured in a humidified chamber of 5% CO_2_ and 95% air at 37 °C.

#### 2.1.1. Osteoblasts

MC3T3-E1 cells, a murine pre-osteoblast line (Dr. Noman Karin, Roswell Park Cancer Institute, Buffalo, NY, USA), were maintained in growth medium containing alpha Minimum Essential Medium (αMEM) (Gibco, Gaithersburg, MD, USA), 10% FBS (HyClone, Logan, UT, USA), and 1% Penicillin 100 U/mL/Streptomycin 100 μg/mL (Gibco). Twenty-four hours later, cells were rinsed with 1X phosphate buffered saline (PBS; HyClone), and the medium was replaced with 1X differentiation medium (αMEM (Gibco), 10% FBS (HyClone), 1% 100 U/mL penicillin/100 μg/mL Streptomycin (Gibco), 50 μg/mL ascorbic acid (Sigma, St. Louis, MO, USA), and 10 mM β-glycerophosphate (Sigma)) and grown to late differentiation (20 days) [40]. Differentiation medium was changed every third day.

#### 2.1.2. Osteoclast Precursors

RAW 264.7 cells, a murine monocyte/macrophage cell line capable of differentiation into mature osteoclasts with resorptive capabilities [43,44], were generously provided by Dr. Yibin Kang (Princeton University, Princeton, NJ, USA). Cells were maintained in growth medium containing RPMI (Gibco) supplemented with 10% FBS (HyClone) and 1% 100 U/mL penicillin/100 mg/mL streptomycin (Gibco). To differentiate RAW 264.7 cells in-vitro, 50 ng/mL exogenous RANKL (PeproTech, Rocky Hill, NJ, USA) was added to cultures for six days. Media and exogenous RANKL were replaced every second day. RAW 264.7 cells can secrete macrophage colony stimulating factor (M-CSF) on their own, thus, exogenous M-CSF is unnecessary for differentiation [45,46,47]. 

#### 2.1.3. Breast Cancer Cell Variants

MDA-MB-231 human triple-negative metastatic breast cancer cells were derived from a pleural effusion of an adenocarcinoma [48]. MDA-MB-231 breast cancer cells were a gift from Dr. Dan Welch (Kansas University Medical Center, Kansas City, KS, USA). MDA-MB-231 breast cancer cells were maintained in growth medium containing Dulbecco’s Minimal Eagle Medium (DMEM; Gibco) supplemented with 5% FBS (HyClone) and 1% 100 U/mL penicillin/100 mg/mL streptomycin (Gibco).

For in-vivo experiments, MDA-MB-231GFP/luciferase (MDA-MB-231 GFP/luc) breast cancer cells were used. MDA-MB-231GFP/luc breast cancer cells express the green fluorescent protein (GFP) and luciferase (pLe-Go-IG2-Luc2 vector) and were a gift from Dr. Alessandro Fatatis (Drexel University, Philadelphia, PA, USA). MDA-MB-231GFP/luciferase cells (MDA-MB-231 GFP/luc) are equivalent to MDA-MB-231 cells, but have been engineered to express GFP and the Luc2 vector [49]. Cells were maintained in DMEM (Gibco) supplemented with 5% FBS (HyClone) and 10 mg/mL Gentamycin (Thermo Fisher Scientific, Waltham, MA, USA).

MCF-7 human ER+ breast cancer cells were derived from a pleural effusion [50] and were purchased directly from the ATCC (Manassas, VA, USA). Cells were maintained in growth medium containing DMEM (Gibco) supplemented with 10% FBS (HyClone), 1% 100 U/mL Penicillin/100 mg/mL Streptomycin (Gibco), and 0.01 μg/mL of recombinant human insulin (MP Biomedicals, Solon, OH, USA).

### 2.2. Breast Cancer Conditioned Media

MDA-MB-231 triple-negative metastatic breast cancer cells were grown until 70–80% confluency. Cells were then rinsed with 1X PBS (HyClone), and serum-free αMEM (Gibco) was added for 24 h. After twenty-four hours, MDA-MB-231 breast cancer conditioned media (MDA-MB-231 CM) was collected, centrifuged to remove cellular debris, and stored at −80 °C until use.

MCF-7 human ER+ breast cancer cells were grown until 70–80% confluency. Cells were then rinsed with 1X PBS (HyClone) and serum-free αMEM (Gibco) was added for 24 h. After 24 h, MCF-7 breast cancer cell conditioned media (MCF-7 CM) was collected, centrifuged to remove cellular debris, and stored at −80 °C until use.

### 2.3. Osteoblast Conditioned Media

MC3T3-E1 cells, grown for 20 days, were rinsed with 1X PBS (HyClone) and serum-free αMEM was added for 24 h. After 24 h, naïve osteoblast conditioned media (naïve OB CM) was collected, centrifuged to remove cellular debris, and stored at −80 °C until use.

### 2.4. Generation of EOs In-Vitro

MC3T3-E1 cells grown to 20 days were rinsed with 1X PBS (HyClone), then exposed to MDA-MB-231 CM or MCF-7 CM to produce “educated” osteoblasts (EOs) in-vitro. The treatment formulation consisted of three parts 1.5X differentiation medium (αMEM (Gibco), 15% FBS (HyClone), 15 mM β-glycerophosphate (Sigma, St. Louis, MO, USA), 75 μg/mL ascorbic acid (Sigma), and 1% 100 U/mL penicillin/100 μg/mL streptomycin (Gibco)) plus 1-part MDA-MB-231 CM or MCF-7 CM for an additional 21 days [40]. Media were changed every second day. EO cells are denoted by the conditioned medium they were exposed to: EO-231 cells (osteoblasts exposed to MDA-MB-231 CM) or EO-MCF-7 cells (osteoblasts exposed to MCF-7 CM).

### 2.5. EO Cell Conditioned Media

EO cells were maintained in EO cell growth media until they reached 70–80% confluency. Cells were then rinsed with 1X PBS (HyClone), and serum-free αMEM (Gibco) was added for 24 h. EO cell conditioned media (EO-231 or EO-MCF-7 CM) were collected, centrifuged to remove cellular debris, and stored at −80 °C until use.

### 2.6. Soluble Protein Expression of Osteoclastogenic Factors RANKL, OPG, and TNFα

EO-231, EO-MCF-7 CM, and naïve OB CM were subjected to sandwich enzyme-linked immunosorbent assay (ELISA) for soluble protein expression of osteoclast-associated factors receptor activator of nuclear factor kappa-B ligand (RANKL), osteoprotegerin (OPG), and tumor necrosis factor alpha (TNFα). All antibodies and protein standards were purchased from R&D Systems (Minneapolis, MN, USA).

Capture antibodies (RANKL capture = 0.8 μg/mL; OPG capture = 8 μg/mL; TNFα capture = 0.8 μg/mL) were diluted in Ngai’s buffer (15 mM Na_2_HCO_3_, pH 9.6) and incubated overnight at 4 °C. After 24 h, wells were rinsed with 1X PBS/Tween and blocked for 2 h at room temperature with 1% bovine serum albumin (BSA, Thermo Fisher Scientific) in 1X sterile PBS. After blocking, plates were washed with 1X PBS/Tween, and conditioned medium samples or standard were added to the respective wells. A standard curve for each protein of interest was prepared fresh each time. Wells were incubated overnight at 4 °C. After 24 h, plates were washed with 1X PBS/Tween, and biotinylated detection antibodies (RANKL detection = 0.4 μg/mL; OPG detection = 0.4 μg/mL; TNFα detection = 0.4 μg/mL) were diluted in 1% BSA in 1X sterile PBS and incubated for 2 h at room temperature. After 2 h, the plates were washed with 1X PBS/Tween and incubated with NeutrAvidin Horseradish conjugate (Thermo Fisher Scientific) diluted in 1X sterile PBS for 30 min at room temperature. After 30 min, plates were washed with 1X PBS/Tween and ABTS substrate (Thermo Fisher Scientific) diluted in 3% hydrogen peroxide was added to plates for 90 min in the dark at room temperature. The plates were read at 405 nm using a spectrophotometer (SpectraMax 190; Molecular Devices, San Jose, CA, USA).

### 2.7. Separation of CD11b+ Mononuclear Cells from Murine Bone Marrow Aspirate

Primary bone marrow monocytes were isolated from the bone marrow of femur and tibiae of four-week-old C57BL/six female mice [51]. Mice were euthanized and femurs and tibiae were harvested and cleaned free of soft tissue. Bone ends were removed, and bone marrow was flushed using αMEM (Gibco) plus 1% 100 U/mL penicillin/100 mg/mL streptomycin (Gibco). A total of 10 femurs and 10 tibiae were flushed. Bone marrow aspirate was plated in osteoblast growth medium (αMEM (Gibco) supplemented with 10% FBS (HyClone) and 1% 100 U/mL penicillin/100 mg/mL streptomycin (Gibco)) for 24 h.

After 24 h, nonadherent cells were harvested by collecting the medium and centrifuging for 10 min at 1000 rpm. Cells were resuspended in 2 mL of 1X PBS plus 5% FBS (HyClone) for separation, as previously described [51]. CD11b+ mononuclear cells were collected using the EasySep™ Mouse CD11b Positive Selection Kit (STEMCELL Technologies, Vancouver, Canada). CD11b+ cells were maintained in a medium of αMEM (Gibco), 10% FBS (HyClone), and 1% 100 U/mL penicillin/100 mg/mL streptomycin (Gibco) plus 10 ng/mL M-CSF (PeproTech, Rocky Hill, NJ, USA) [51]. These CD11b+ cells were used as a source of enriched osteoclast precursors.

### 2.8. Identification of TRAP+ Osteoclasts in Cultures Containing CD11b+ Primary Bone Marrow Monocytes

1 × 10^3^ CD11b+ mononuclear cells were co-cultured with 5 × 10^2^ MC3T3-E1 naïve OBs, EO-231 cells, or EO-MCF-7 cells for six days in osteoclast differentiation medium (αMEM (Gibco), 10% FBS (HyClone), and 1% 100 U/mL penicillin/100 μg/mL streptomycin (Gibco)), supplemented with 10 ng/mL exogenous M-CSF (PeproTech) and 7.5 ng/mL RANKL (PeproTech). Media, exogenous M-CSF, and exogenous RANKL were replaced every second day. After six days, cultures were fixed with 10% neutral buffered formalin (Thermo Fisher Scientific) for 10 min. After 10 min, cultures were rinsed with deionized water and stained for TRAP to identify TRAP+, multinucleated osteoclasts (≥3 nuclei). TRAP staining solution was composed of: (a) 200 mL TRAP basic incubation medium (1 L total volume; pH 4.9, composed of (i) 9.2 g sodium acetate anhydrous (Sigma); (ii) 11.4 g L-(+) tartaric acid (Sigma); (iii) 2.8 mL glacial acetic acid (Sigma); and (iv) 950 mL deionized water), (b) 1 mL Naphthol AS-MX Phosphate substrate mix (20 mg/mL in Ethylene glycol monoethyl ether (Research Products International, Mount Prospect, IL, USA)) and (c) Fast Red Violet LB Salt (120 mg; Sigma). Wells were incubated with TRAP stain for 7 min at 37 °C, washed with deionized water, and then imaged using a light microscope. The number of TRAP+, multinucleated osteoclasts were quantified per condition.

### 2.9. Identification of TRAP+ Osteoclasts in Cultures Containing RAW 264.7 Pre-Osteoclasts

#### 2.9.1. Conditioned Medium

Tartrate resistance acid phosphatase (TRAP) staining is a long-established way to identify mature osteoclasts in-vitro [52]. 1 × 10^4^ RAW 264.7 pre-osteoclasts were exposed to naïve OB CM, EO-231 CM, or EO-MCF-7 CM in the presence of 50 ng/mL exogenous RANKL (PeproTech) for six days. The treatment formulation consisted of three parts osteoclast differentiation medium (αMEM (Gibco), 10% FBS (HyClone), and 1% 100 U/mL penicillin/100 μg/mL streptomycin (Gibco)) plus one-part naïve OB CM (control), EO-231 CM, or EO-MCF-7 CM. 1 × 10^4^ RAW 264.7 pre-osteoclasts in osteoclast differentiation medium in the presence of 50 ng/mL exogenous RANKL served as an additional control. Media and exogenous RANKL were replaced every second day. After six days, wells were fixed with 10% neutral buffered formalin (Thermo Fisher Scientific) for 10 min. Cultures were then rinsed with deionized water and stained using the acid phosphatase, leukocyte (TRAP) staining kit protocol (Sigma) to identify TRAP+ osteoclasts and imaged using a light microscope. The number of TRAP+, multinucleated (≥3 nuclei) osteoclasts were quantified per condition. At least three individual replicates were imaged and quantified per condition.

#### 2.9.2. Co-Culture

1 × 10^4^ RAW 264.7 pre-osteoclasts were co-cultured with 1 × 10^3^ MC3T3-E1 naïve OBs, EO-231 cells, or EO-MCF-7 cells in the presence of 50 ng/mL exogenous RANKL (PeproTech) for six days. 1 × 10^4^ RAW 264.7 pre-osteoclasts in osteoclast differentiation medium in the presence of 50 ng/mL exogenous RANKL served as an additional control. Media and exogenous RANKL were replaced every second day. After six days, wells were fixed with 10% neutral buffered formalin (Thermo Fisher Scientific) for 10 min. Cultures were then rinsed with deionized water and stained using the acid phosphatase, leukocyte (TRAP) staining kit protocol (Sigma) to identify TRAP+ osteoclasts and imaged using a light microscope. The number of TRAP+, multinucleated (≥3 nuclei) osteoclasts were quantified per condition. At least three individual replicates were imaged and quantified per condition.

### 2.10. In-Vitro Bone Resorption Assay and Quantification on a Bone Mimetic Matrix

#### 2.10.1. Identification and Quantification of TRAP+ Osteoclasts on a Bone Mimetic Matrix

1 × 10^4^ RAW 264.7 pre-osteoclasts were co-cultured with 1 × 10^3^ MC3T3-E1 naïve OBs, EO-231 cells, or EO-MCF-7 cells in the presence of 50 ng/mL exogenous RANKL (PeproTech) on 24 well plates coated with a bone mimetic, synthetic matrix (OsteoAssay Surface Plate; Corning, Corning, NY, USA) for six days. 1 × 10^4^ RAW 264.7 pre-osteoclasts in osteoclast differentiation medium in the presence of 50 ng/mL exogenous RANKL cultured on a bone mimetic synthetic matrix served as an additional control. After six days, wells were fixed with 10% neutral buffered formalin (Thermo Fisher Scientific) for 10 min and stained with TRAP to identify TRAP+ osteoclasts. The TRAP solution was composed of (a) 50 mL 0.1 M acetate buffer (35.2 mL 0.2 sodium acetate solution (16.4 g/L sodium acetate; Fisher) in water and 14.8 mL 0.2 acetic acid solution (11.5 mL glacial acetic acid (Fisher) in 988.5 mL water)), (b) 10 mL 0.3 M sodium tartrate (6.9 g (Sigma) per 100 mL water), (c) 100 ul Triton X-100 (Fisher), and (d) water for a total of 1 L TRAP buffer solution. The 50 mL TRAP buffer was warmed to 37 °C, and 1 mL 10 mg/mL Napthol AS-MX phosphate (Thermo Fisher Scientific) and 0.3 mg Fast Red violet LB salt per ml were added to the TRAP buffer to make the TRAP staining solution. Five hundred µl of TRAP staining solution was added to individual wells, and then incubated for 10 min at 37 °C. After 10 min, TRAP buffer was removed, wells were washed with deionized water and imaged using a light microscope. The number of TRAP+, multinucleated osteoclasts were quantified per condition. At least three individual replicates were imaged and quantified per condition.

#### 2.10.2. Identification and Quantification of Osteoclast Resorptive Pits on Bone Mimetic Matrix

TRAP-stained cells were released from the bone mimetic synthetic matrix using 5% bleach in water for 7 min at room temperature. Bleach was slowly removed from wells, and wells were rinsed with deionized water. Pictures were taken of resorptive pits using a light microscope. Twenty pictures were taken and analyzed per well for each condition. Images were captured as tagged image format files (TIF) files. The resorption pit perimeters were traced using Paint (Version 2004, Windows, Redmond, WA, USA). The TIF files were then converted to portable image graphic files (PNG) using Adobe Photoshop (Version 20.0.6). The PNG files were imported to SketchandCalc software (www.sketchandcalc.com), and resorptive pit areas were then calculated using the SketchandCalc magic wand tool, which fills in the outlined perimeter. The total area of the section was determine using the SketchandCalc rectangle tool to outline the total section. The percent area was determined for each pit by dividing the pit’s area by the total section area and multiplying this result by 100.

Additionally, the wells were stained using a modified von Kossa staining protocol. This protocol is used to improve the contrast of resorbed pit visualization and analysis. For the modified von Kossa staining, 5% aqueous silver nitrate was added to each of the wells and incubated in the dark at room temperature for 30 min. After 30 min, wells were rinsed with deionized water, and wells were air dried. The remaining mineral matrix appears a dark yellow color. Pictures were taken of resorptive pits using a light microscope. At least five images were taken and analyzed per well for each condition. Image analysis was performed using Olympus cellSens Count and Measure analysis software (version 1.18).

### 2.11. Intratibial Inoculations and TRAP Stain of Murine Bone Sections

#### 2.11.1. Intratibial Inoculation of Murine Tibiae

MDA-MB-231GFP/Luc2 cells, MC3T3-E1 naïve OBs, and EO 231 cells were grown to 70–80% confluency. Cells were individually collected and resuspended in 1X PBS (HyClone). MDA-MB-231GFP/Luc2 cells were admixed with MC3T3-E1 naïve OBs or EO 231 cells at a 2:1 ratio, respectively, whereby a total of 5 × 10^5^ cells total in 10 μL PBS were injected into the tibiae of female athymic mice aged 5–6 weeks (Harlan Sprague-Dawley, Indianapolis, IN, USA). MDA-MB-231 GFP/Luc2 cells inoculated alone served as controls (5 × 10^5^ cells in 10 μL PBS). Briefly, mice were anesthetized via an intraperitoneal injection of a mixture of ketamine (129 mg/kg) and xylazine (4 mg/kg). Once the mice were fully anesthetized as evidenced by a toe pinch and lack of movement, for intratibial injections, the hind leg was bent to a 90° position and 27-gauge needle with cells inserted through the patellar tendon and into the proximal tibia using gentle pressure and twisting motion [53]. At least four mice were utilized per experimental group. IVIS Imaging (Perkin Elmer, Waltham, MA, USA) was used to monitor tumor formation for luciferase expression. Mice were maintained under the guidelines of the NIH and Thomas Jefferson University. All protocols were approved and monitored by the Institutional Animal Care and Use Committee.

#### 2.11.2. Removal of Murine Tibiae and Bone Preparation

Mice were euthanized via CO_2_ inhalation followed by cervical dislocation once tumors reached an average radiance (p/s/cm^2^/sr) of 1 × 10^8^. Tibiae were harvested and fixed for 24 h in 4% paraformaldehyde (Electron Microscopy Sciences, Hatfield, PA, USA) at 4 °C. After 24 h, fixed tibiae were decalcified for an additional 48 h with 0.5 mol/l EDTA in dH2O (Sigma) at 4 °C. Bones were then embedded in 30% sucrose in 1X PBS for 24 h and placed in Shandon CyromatrixTM embedding medium (Thermo Shandon, Waltham, MA, USA) and snap frozen in liquid nitrogen [40]. Frozen samples were wrapped in aluminum foil and stored at −80 °C. Frozen samples were cryosectioned at ten-micron-thick using a Diamond High Profile Knife (C.L. Sturkey, Lebanon, PA, USA) on a Leica CM3050 Cryostat (Leica, Inc., Nussloch, Germany). Pre-chilled adhesive transfer tape windows (Leica Inc., Buffalo Grove, IL, USA) were used to transfer cut bone sections onto pre-chilled adhesive-coated slides (CJX adhesive-coated slides; Leica Inc., Buffalo Grove, IL, USA). Two bone serial sections were placed on slides, and transfer tape was removed from slides. Bone sections were permanently bonded to slides after exposure to ultraviolet light for 30 min. Bone sections were stored in slide boxes at −20 °C until use.

#### 2.11.3. TRAP Stain on Murine Tibiae Sections

Slides were removed from freezer and thawed at room temperature for 30 min. Slides were rehydrated using deionized water and incubated with TRAP stain (Section 2.10.1) for 10 min at 37 °C, washed with deionized 1X TBS, and a coverslip was placed on the slide. Slides were then imaged using a Nikon E800 light microscope.

### 2.12. Western Blotting

Cells were lysed in ice-cold RIPA lysis buffer containing 20 mM Tris-HCl (pH 7.4, Sigma), 1% NP-40 (*v*/*v,* Thermo Fisher Scientific), 0.25% Na-deoxycholate (*v*/*v,* Sigma), 150 mM NaCl (Sigma), 1 mM EDTA (Sigma), 1 mM PMSF (Sigma), 1 mM Na_3_VO_4_ (Sigma), and 1 mM NaF (Sigma) plus Halt™ Protease and Phosphatase Inhibitor Cocktail (Thermo Scientific), then gently agitated for 1–2 h at 4 °C. Lysates were centrifuged for 20 min at 14,000 rpm at 4 °C, quantified using *DC™* Protein Assay (Bio-Rad, Hercules, CA), and boiled with loading buffer for 12 min at 95 °C. Proteins were loaded onto a 12% SDS-PAGE gel (Bio-Rad) and separated by running at 100 V for 1 h 30 min. Separated proteins were transferred to 0.45 µm PVDF membranes (Millipore Sigma, Billerica, MA, USA) and blocked for 1 h using SuperBlock Blocking buffer (Thermo Fisher Scientific) or 5% milk in 0.5% TBS/Tween. Membranes were then incubated with primary antibody (monoclonal mouse anti-DC-STAMP (0.5 ug/mL in superblock; Millipore Sigma) or monoclonal mouse anti-β-actin (1:5000 in 5% milk; Sigma)) overnight at 4 °C. Blots were washed in 0.5% TBS/Tween for 1 h, and a secondary antibody goat anti-mouse HRP (1:7500 for DC-STAMP in superblock or 1:5000 for β-actin in 5% milk; Cell Signaling Technology, Danvers, MA, USA) was used. Blots were then washed with 0.5% TBS/Tween for 1 h. Signals were detected using SuperSignal™ West Femto Chemiluminescent Substrate detection kit (Thermo Scientific) and imaged using the Bio-Rad ChemiDoc™ MP Imaging System (Bio-Rad, Hercules, CA, USA). Band densitometry was calculated using ImageJ software.

### 2.13. TNFα Rescue and Neutralization on Pre-Osteoclasts Exposed to EO CM

#### 2.13.1. TNFα Rescue

To examine the effect of TNFα on osteoclast formation, 1 × 10^4^ RAW 264.7 pre-osteoclasts were exposed to naïve OB CM (control), EO-231 CM, and EO-MCF-7 CM in the presence of 50 ng/mL exogenous RANKL (PeproTech) for six days. The treatment formulation consisted of three parts osteoclast differentiation medium (αMEM (Gibco), 10% FBS (HyClone), and 1% 100 U/mL penicillin/100 μg/mL streptomycin (Gibco)) plus one-part naïve OB CM (control), EO-231 CM, or EO-MCF-7 CM. Cultures containing EO CM were supplemented with 12 ng/mL recombinant TNFα protein (R&D Systems). TNFα recombinant protein concentration was based on the difference between naïve OB CM TNFα soluble protein concentration compared to EO-231 CM TNFα soluble protein concentration and EO-MCF-7 CM TNFα soluble protein concentration. 1 × 10^4^ RAW 264.7 pre-osteoclasts in osteoclast differentiation medium in the presence of 50 ng/mL exogenous RANKL served as an additional control. After six days, wells were fixed with 10% neutral buffered formalin (Thermo Fisher Scientific) for 10 min, stained for TRAP, and imaged using a light microscope. The number of TRAP+, multinucleated (≥3 nuclei) osteoclasts were quantified per condition. At least three individual replicates were imaged and quantified per condition.

#### 2.13.2. TNFα Neutralization

1 × 10^4^ RAW 264.7 cells were differentiated with 50 ng/mL soluble RANKL in the presence of vehicle media (VM; control), naïve OB CM (control), EO-231 CM, or EO-MCF-7 CM. The treatment formulation consisted of three parts osteoclast differentiation media (αMEM (Gibco), 10% FBS (HyClone), and 100 U/mL penicillin/100 μg/mL streptomycin (Gibco)), plus one-part OB CM, EO-231 CM, or EO-MCF-7 CM in the presence of 50 ng/mL RANKL for six days. Cultures containing EO CM were supplemented with 12 ng/mL recombinant TNFα protein (R&D Systems) plus 2 or 4 μg/mL anti-TNFα (R&D Systems). As an additional control, RAW 264.7 pre-osteoclasts were differentiated with 50 ng/mL soluble RANKL in the presence of EO-231 CM or EO-MCF-7 CM supplemented with 12 ng/mL recombinant TNFα protein (R&D Systems) plus 2 or 4 µg/mL polyclonal IgG antibody (R&D Systems). After six days, wells were fixed with 10% formalin and stained for TRAP. Wells were imaged using light microscopy. The number of TRAP+, multinucleated (≥3 nuclei) osteoclasts were quantified per condition. At least three individual replicates were imaged and quantified per condition.

### 2.14. Statistical Analysis

Statistical analyses were carried out using GraphPad Prism 8 (GraphPad, La Jolla, CA, USA). For all analyses, one-way ANOVA with Tukey’s multiple comparisons test was used. Significance was defined at a two-sided alpha level of 0.05.

## 3. Results

### 3.1. EO Cells or Their Conditioned Media Reduce Osteoclast Maturation

It is well established that osteoblasts interact with osteoclast progenitors during osteoclastogenesis [23]. Osteoblasts secrete osteoclast differentiation factors M-CSF and RANKL, as well as RANKL independent factors TGF-β and TNF-α, to stimulate osteoclast progenitor differentiation and promote mature osteoclast formation [10,29,54] (Figure 1). Previously, we have demonstrated that EO cells have altered properties compared to naïve osteoblasts [40], but it is unknown how EO cells affect osteoclastogenesis. The RAW 264.7 cell line was used to assess osteoclast formation. Previous studies have demonstrated that RAW 264.7 pre-osteoclasts cultured in the presence of RANKL begin to fuse and form multinucleated (≥3 nuclei) osteoclasts in the absence of M-CSF [45,46,47] at day 3, with optimal mature osteoclast formation occurring between days 5–7 [54,55]. Therefore, we chose a six-day timepoint to identify mature, multinucleated (≥3 nuclei) osteoclasts.

**Figure 2:**
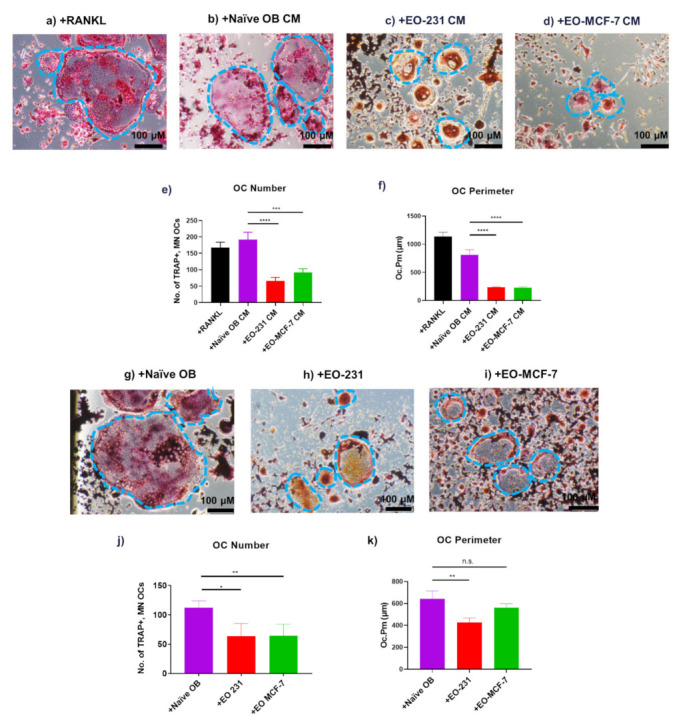
Osteoclasts Produced in the Presence of EO CM or EO Cells are Smaller and Less in Number. RAW 264.7 pre-osteoclasts were culture in the presence of (**a**) 50 ng/mL exogenous RANKL for six days (control) or exposed to the following conditions in the presence of 50 ng/mL exogenous RANKL: (**b**) naïve OB CM (control), (**c**) EO-231 CM, or (**d**) EO-MCF-7 CM. The number (No.) of TRAP+, multinucleated (≥3 nuclei) mature osteoclasts (pink/red/purple color; blue outlines) and osteoclast perimeter (Oc.Pm) were quantified for each CM condition (**e,f**). In a separate experiment, RAW264.7 cells were co-cultured in the presence of 50 ng/mL exogenous RANKL for six days with (**g**) naïve osteoblasts (control), (**h**) EO-231, or (**i**) EO-MCF-7 cells. Cultures were stained for tartrate-resistant acid phosphatase (TRAP), a common maker for mature osteoclasts. The number (No.) of TRAP+, multinucleated mature osteoclasts (pink/red/purple color; blue outlines), and osteoclast perimeter (Oc.Pm) were quantified for co-culture conditions (**j**,**k**). Scale = 100 µM. *N* = 3. * *p* < 0.05; ** *p* < 0.01; *** *p* < 0.001, **** *p* < 0.0001; n.s. = not significant.

To determine how EO cells affect osteoclast maturation, RAW 264.7 pre-osteoclasts were exposed to EO cell conditioned media (i.e., EO-231 CM or EO-MCF-7 CM) in the presence of exogenous RANKL for six days (Figure 2c,d). RAW 264.7 pre-osteoclasts cultured in the presence of exogenous RANKL or exposed to naïve OB CM in the presence of exogenous RANKL for six days served as controls (Figure 2a,b). Mature osteoclasts formed in-vitro are characterized by their large size and expression of tartrate resistant acid phosphatase (TRAP) [52,56]. After six days of treatment, cultures were fixed and stained for TRAP. TRAP is an enzyme secreted by osteoclasts during bone resorption and has been used as an established marker for osteoclasts for more than 50 years [57]. We observed that osteoclasts formed upon exposure to EO CM were smaller in size (Figure 2c,d,f) compared to osteoclasts formed upon exposure to naïve OB CM (Figure 2b,f).

We also quantified the number of TRAP-positive, multinucleated (≥3 nuclei) osteoclasts and found a two-fold reduction in the number of TRAP+, multinucleated (≥3 nuclei) osteoclasts exposed to EO-231 CM or EO-MCF-7 CM when compared to the number of TRAP+, multinucleated (≥3 nuclei) osteoclasts exposed to naïve OB CM (Figure 2e). In addition to osteoclast number, we also quantified the number of multinucleated (≥3 nuclei), binucleated, and mononucleated osteoclasts per condition (Figure S1). We demonstrated that the majority of osteoclasts (>75%) are multinucleated (≥3 nuclei) for all conditions (Figure S1). Additionally, we found a 3.5-fold reduction in the perimeter of osteoclasts exposed to EO CM when compared to the perimeters of osteoclasts exposed to naïve OB CM (Figure 2f). These results suggest EOs secrete soluble factors that alter the formation of osteoclasts in-vitro.

Since we found alterations in osteoclast formation upon exposure to EO CM, we wanted to determine how this affect may be modulated by direct cellular contact. Therefore, RAW 264.7 pre-osteoclasts were co-cultured with naïve osteoblasts (control) or EO cells (i.e., EO-231 or EO-MCF-7) (Figure 2g–i). All conditions were cultured in the presence of exogenous RANKL for six days and stained for TRAP to identify the number of TRAP-positive, multinucleated (≥3 nuclei) osteoclasts. We observed that osteoclasts produced in the presence of EO cells were smaller in size (Figure 2h,i,k) than osteoclasts produced in the presence of naïve osteoblasts (Figure 2g,k). Similar to results observed when osteoclasts were produced in the presence of EO CM, we found a 30% reduction in the number of TRAP-positive, multinucleated (≥3 nuclei) osteoclasts produced in the presence of EO-231 cells and a 19% reduction in the number of TRAP-positive, multinucleated (≥3 nuclei) osteoclasts produced in the presence of EO-MCF-7 cells when compared to the number of osteoclasts produced in the presence of naïve osteoblasts (Figure 2j). Osteoclasts produced in the presence of EO-231 cells had 33% smaller perimeters compared to those produced in the presence of naïve osteoblasts (Figure 2k). Although not significant, we found osteoclasts produced in the presence of EO-MCF-7 cells were 12% smaller than osteoclasts produced in the presence of naïve osteoblasts (Figure 2k). These results suggest that direct interaction between EO cells and osteoclast precursors reduce the number of TRAP-positive osteoclasts formed in-vitro.

We next utilized CD11b+ primary bone marrow monocytes (BMMs) to assess osteoclast maturation in vitro [58,59]. CD11b is a part of an integrin complex with CD18 [60]. Integrins are important to adhesion, trafficking, and differentiation of cells, especially for monocytes/macrophages [59]. The CD11b/CD18 integrin complex and the CD11a/CD18 integrin complex are expressed on osteoclast precursors and play important roles in osteoclast differentiation and downstream activation of osteoclast pathways [59]. Multiple studies have found that CD11b precursors, also known as myeloid-suppressor cells, are abundantly found in the bone marrow and have overlapping lineage with osteoclasts [59,61,62,63], making them ideal candidates for osteoclast differentiation.

CD11b+ primary BMMs were co-cultured with naïve osteoblasts (control), EO-231 cells, or EO-MCF-7 cells in the presence of exogenous M-CSF and exogenous RANKL for six days (Figure S2) [64]. After six days, cultures were stained for TRAP and the number of TRAP-positive, multinucleated (≥3 nuclei) osteoclasts were quantified for each condition. CD11b+ primary BMMs formed TRAP-positive, multinucleated (≥3 nuclei) osteoclasts in the presence of EO cells (Figure S2b,c) or naïve osteoblasts (Figure S2a) with the addition of exogenous M-CSF and exogenous RANKL.

Additionally, we found a 2.4-fold reduction in the number of TRAP-positive, multinucleated (≥3 nuclei) osteoclasts produced in the presence of EO-231 cells and a two-fold reduction in the number of TRAP-positive, multinucleated (≥3 nuclei) osteoclasts produced in the presence of EO-MCF-7 cells when compared to the number of osteoclasts produced in the presence of naïve osteoblasts (Figure S2d). These results corroborate our earlier findings with the RAW 264.7 pre-osteoclast cell line, where we found that osteoclasts produced in the presence of EO cells are less in number (Figure 2j). These data confirm EO cells’ suppressive effect on osteoclastogenesis.

### 3.2. Osteoclasts Produced in the Presence of EO Cells Have Reduced Resorption

Next, we wanted to determine how EO cells affect osteoclast resorption. Mature osteoclasts are large, multinucleated (≥3 nuclei), TRAP-positive cells [65]. Mature, active osteoclasts are further characterized by a ruffled border membrane [66] and formation of a resorptive pit in-vitro [67]. During bone resorption, osteoclasts bind to the bone matrix through the ruffled border membrane, creating a “sealing zone”, where lysosomal enzymes, such as TRAP, are released into the resorptive pit [23,68]. The resorptive pit is the sealed area where bone is resorbed [56,69]. Since we observed decreased osteoclast formation when EO cells or their CM were present, we hypothesized that osteoclasts produced in the presence of EO cells would have decreased resorptive activity.

**Figure 3:**
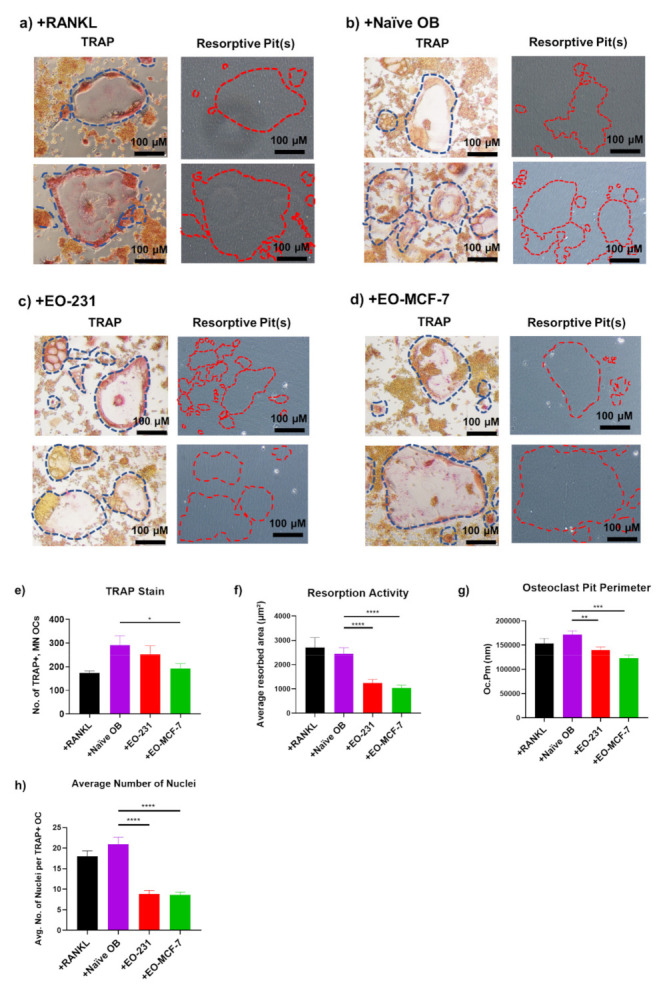
Osteoclasts produced in the presence of EO cells have decreased resorptive activity and reduced pit perimeter. RAW 264.7 pre-osteoclasts were co-cultured on a bone mimetic matrix with either (**a**) 50 ng/mL RANKL (control), or in the presence of 50 ng/mL RANKL under the following conditions: (**b**) naïve OBs (control), (**c**) EO-231, or (**d**) EO-MCF-7. After six days, cultures were stained for TRAP (pink color; blue outlines), a common marker for mature osteoclasts. Formation of resorptive pits were also observed (red outlines) and matched to the corresponding TRAP picture for each condition. Representative images are displayed per condition. The number (No.) of TRAP-positive, multinucleated (≥3 nuclei) mature osteoclasts (**e**), resorptive area (**f**), osteoclast pit perimeter (Oc.Pm) (**g**), and the number of nuclei per TRAP-positive, multinucleated (≥3 nuclei) osteoclast (**h**) were quantified for each condition. Three individual batches were assayed per condition. TRAP and resorptive pits were visualized via light microscopy. Scale = 100 µM. *N* = 3. * *p* < 0.05; ** *p* < 0.01; *** *p* < 0.001; **** *p* < 0.0001.

To determine how EO cells affect osteoclast resorption, RAW 264.7 pre-osteoclasts were co-cultured on a bone mimetic surface with naïve osteoblasts (control), EO-231 cells, or EO-MCF-7 cells in the presence of exogenous RANKL for six days. As an additional control, RAW 264.7 pre-osteoclasts alone were cultured on a bone mimetic surface in the presence of exogenous RANKL for six days. After six days, cultures were first stained for TRAP and imaged via light microscopy (Figure 3a–d).

We found a 34% reduction in the number of TRAP-positive, multinucleated (≥3 nuclei) osteoclasts produced in the presence of EO-MCF-7 cells on a bone-like matrix when compared to the number of TRAP-positive, multinucleated (≥3 nuclei) osteoclasts produced in the presence of naïve osteoblasts (Figure 3e). Although not significant, we additionally saw a 13% reduction in the number of TRAP-positive, multinucleated (≥3 nuclei) osteoclasts produced in the presence of EO-231 cells cultured on a bone-like matrix compared to the number of TRAP-positive, multinucleated (≥3 nuclei) osteoclasts produced in the presence of naïve osteoblasts (Figure 3e).

Immediately after imaging, the cells were removed from the bone-like matrix to observe the resorptive pits underneath. To our knowledge, this is the first time a simultaneous TRAP and resorptive pit analysis on the same bone-like matrix has been completed. Osteoclast function was determined by quantifying the resorbed area per osteoclast (Figure 3f) and the pit perimeter per osteoclast (Figure 3g) per condition. To further increase the contrast between the bone-like matrix and the resorptive pits, we stained the remaining matrix using a modified von Kossa stain protocol to improve the contrast between the resorptive pits and the remaining bone-like matrix (Figure S3a–d). We then quantified the resorbed area per osteoclast per condition (Figure S3e). Importantly, we found that osteoclasts formed in the presence of EO-231 cells or EO-MCF-7 cells resorbed significantly less bone-like matrix, about 50% less, compared to osteoclasts formed in the presence of naïve osteoblasts cultured on a bone-like matrix (Figure 3f). These results were corroborated by modified von Kossa stain (Figure S3e). We also estimated the resorptive activity of an individual osteoclast by dividing the average resorbed area (Figure 3f) by the average number of TRAP-positive, multinucleated (≥3 nuclei) osteoclasts (Figure 3e) to get an estimation of the resorptive activity of an individual osteoclast for each condition (Table S1). These results demonstrated that an individual osteoclast produced in the presence of EO cells resorbs less bone-like matrix compared to an individual osteoclast produced in the presence of naïve osteoblasts (approximately 30% less bone-like matrix) or an individual osteoclast produced in the presence of exogenous RANKL (approximately 3-fold less bone-like matrix) (Table S1), which corroborates our earlier findings.

Additionally, osteoclasts produced in the presence of EO-231 cells or EO-MCF-7 cells cultured on the bone-like matrix had significantly decreased pit perimeter compared to osteoclasts produced in the presence of naïve osteoblasts cultured on the bone-like matrix (Figure 3g). We also quantified the number of nuclei per TRAP-positive osteoclast and found that osteoclasts produced in the presence of EO cells have, on average, approximately 50% less nuclei compared to TRAP-positive osteoclasts that are produced in the presence of naïve osteoblasts or exogenous RANKL (Figure 3h). These data suggest that osteoclasts produced in the presence of EO cells are functionally active yet are smaller and resorb less bone-like matrix compared to osteoclasts produced in the presence of naïve osteoblasts.

### 3.3. The Number of Osteoclasts Produced in the Presence of EO Cells Plus Breast Cancer Cells Are Reduced

Breast cancer cells that metastasize to bone express and secrete a vast array of proteins and growth factors known to stimulate osteoclast differentiation and maturation [38]. As part of the well-described “vicious cycle”, breast cancer cells secrete factors such as parathyroid hormone related protein (PTHrP), which stimulates osteoblasts to secrete increased amounts of RANKL [35]. This indirect activation of osteoclastogenesis by breast cancer cells causes an increase in osteoclast formation and activation, leading to increased bone destruction and overall bone loss [38]. Osteoclast degradation of bone causes the release of growth factors and proteins stored in the bone-matrix and fuels this feed-forward cycle [3]. Knowing this, we wanted to determine how breast cancer cells regulate osteoclastogenesis in the presence of EO cells.

**Figure 4:**
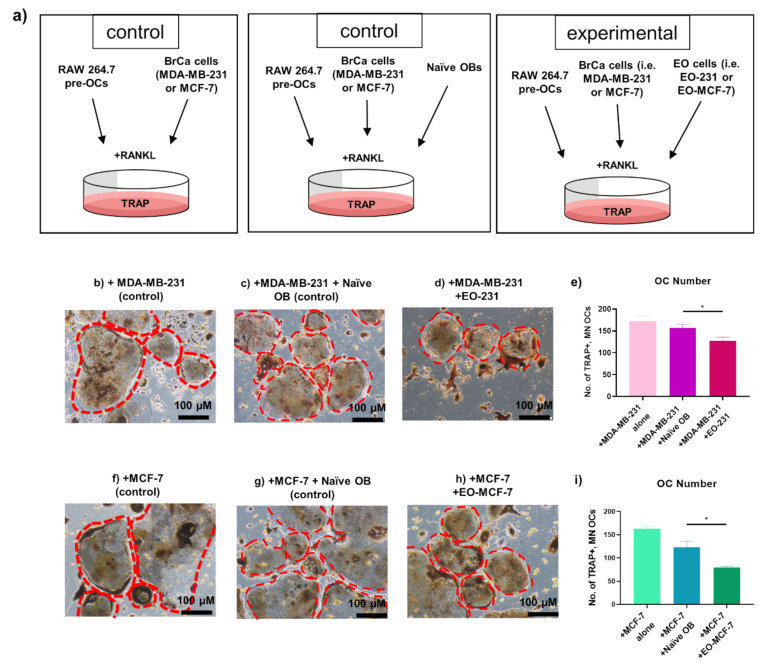
EOs Decrease the Number of TRAP+, Multinucleated Osteoclasts in the Presence of Breast Cancer Cells. (**a**) Cartoon of experimental design. As controls, RAW 264.7 pre-osteoclasts were co-cultured with either MDA-MB-231 human breast cancer cells in the presence of 50 ng/mL exogenous RANKL alone for six days or MCF-7 human breast cancer cells in the presence of 50 ng/mL exogenous RANKL alone for six days (first panel). As additional controls, RAW 264.7 pre-osteoclasts were co-cultured in the presence of 50 ng/mL RANKL for six days with either MDA-MB-231 or MCF-7 human breast cancer cells plus naïve osteoblasts (second panel). The experimental condition included RAW 264.7 pre-osteoclasts co-cultured with MDA-MB-231 human breast cancer cells plus EO-231 cells in the presence of 50 ng/mL exogenous RANKL for six days or MCF-7 human breast cancer cells plus EO-MCF-7 cells in the presence of 50 ng/mL exogenous RANKL for six days (third panel). After six days, the following conditions containing MDA-MB-231 human breast cancer cells were TRAP stained and imaged to identify mature osteoclast formation: (**b**) RAW 264.7 pre-osteoclasts co-cultured with MDA-MB-231 human breast cancer cells in the presence of 50 ng/mL exogenous RANKL alone; (**c**) RAW 264.7 pre-osteoclasts co-cultured with MDA-MB-231 cells plus naïve osteoblasts in the presence of 50 ng/mL exogenous RANKL or (**d**) RAW 264.7 pre-osteoclasts co-cultured with MDA-MB-231 cells plus EO-231 cells in the presence of 50 ng/mL exogenous RANKL. (**e**) The number (No.) of TRAP-positive, multinucleated (≥3 nuclei) mature osteoclasts were quantified for conditions containing MDA-MB-231 breast cancer cells. After six days, the following conditions containing MCF-7 human breast cancer cells were TRAP stained and imaged: (**f**) RAW 264.7 pre-osteoclasts co-cultured with MCF-7 human breast cancer cells in the presence of 50 ng/mL exogenous RANKL alone; (**g**) RAW 264.7 pre-osteoclasts co-cultured with MCF-7 cells plus naïve osteoblasts in the presence of 50 ng/mL RANKL; or (**h**) RAW 264.7 pre-osteoclasts co-cultured with MCF-7 cells plus EO-MCF-7 cells in the presence of 50 ng/mL RANKL. The number (No.) of TRAP-positive, multinucleated (≥3 nuclei) mature osteoclasts were quantified for conditions containing MCF-7 breast cancer cells (**i**). Representative images are shown. Scale = 100 µM. *N* = 3–5. * *p* < 0.05.

To assess for alterations in osteoclast formation, RAW 264.7 pre-osteoclasts were tri-cultured with A) MDA-MB-231 human triple negative breast cancer cells (i) plus naïve osteoblasts (control) or (ii) plus EO-231 cells; or with B) MCF-7 human ER+ breast cancer cells (i) plus naïve osteoblasts (control) or (ii) plus EO-MCF-7 cells all in the presence of exogenous RANKL for six days (Figure 4a). As additional controls, RAW 264.7 pre-osteoclasts were co-cultured in the presence of exogenous RANKL with (i) MDA-MB-231 human triple negative breast cancer cells alone or (ii) MCF-7 human ER+ breast cancer cells alone (Figure 4a). We specifically chose breast cancer cell subtypes that are most representative of human disease, i.e., triple-negative (MDA-MB-231) and ER+ (MCF-7). After six days, all cultures were subjected to TRAP stain and imaged (Figure 4b–d,f–h). The number of TRAP-positive, multinucleated (≥3 nuclei) osteoclasts were quantified per condition (Figure 4e,i).

We observed a 19% reduction in the number of TRAP-positive, multinucleated (≥3 nuclei) osteoclasts produced in the presence of EO-231 cells plus MDA-MB-231 breast cancer cells when compared to the number of TRAP-positive, multinucleated (≥3 nuclei) osteoclasts produced in the presence of naïve osteoblasts plus MDA-MB-231 breast cancer cells (Figure 4e). We also observed a 36% reduction in the number of TRAP-positive, multinucleated (≥3 nuclei) osteoclasts produced in the presence of EO-MCF-7 cells plus MCF-7 breast cancer cells when compared to the number of TRAP-positive, multinucleated (≥3 nuclei) osteoclasts produced in the presence of naïve osteoblasts plus MCF-7 breast cancer cells (Figure 4i). These data provide evidence to suggest that EO cells reduce osteoclastogenesis in the presence of breast cancer cells.

### 3.4. Osteoclast Size Is Decreased When EO Cells Are Present in the Niche

It is well established that breast cancer cells interact with osteoblasts and osteoclasts and utilize the plethora of cytokines, chemokines, and growth factors found in the bone-tumor microenvironment to fuel cancer progression and bone degradation [20,70]. We have previously shown that osteoclasts produced in the presence of EOs are smaller, less in number, and have reduced resorptive activity in-vitro. Therefore, we next wanted to determine how EO cells affect osteoclastogenesis in a physiological setting.

**Figure 5:**
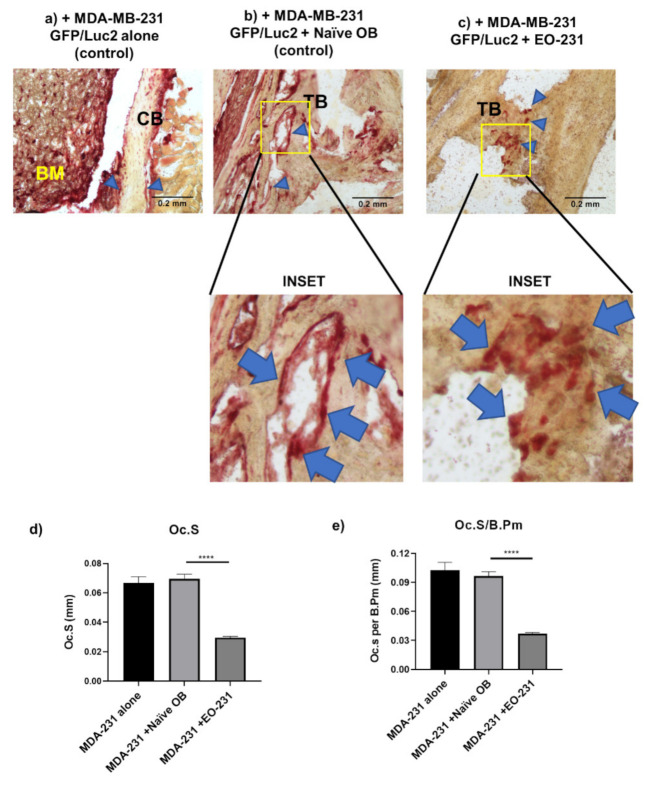
Increased Presence of EOs in Murine Bone Reduces Presence of TRAP-Positive Osteoclasts. Six-week-old female athymic nude mice were injected via the intratibial route with either one of the following: (**a**) human triple negative MDA-MB-231 GFP/Luc2 breast cancer cells alone (control); (**b**) naive osteoblasts plus human triple negative MDA-MB-231 GFP/Luc2 breast cancer cells (control); or (**c**) EO-231 cells plus human triple negative MDA-MB-231 GFP/Luc2 breast cancer cells. The mice were sacrificed eight weeks post-injection, when their tibiae were harvested, sectioned, then stained for the presence of tartrate-resistant acid phosphatase (TRAP; blue arrows), a common marker for mature osteoclasts. One representative image is displayed per condition. (**d**) The surface length (Oc.S) of TRAP-positive, mature osteoclasts were quantified for each condition. Additionally, (**e**) the surface size (Oc.S) of TRAP-positive, mature osteoclasts per bone perimeter (B.Pm) were quantified for each condition. Scale = 0.2. *N >* 5. mm = millimeter. BM, bone marrow; CB, cortical bone; TB, trabecular bone. **** *p* < 0.0001.

To assay for osteoclast formation in-vivo (Figure 5), we injected female athymic nude mice via the intratibial route with either one of the following: (a) naive osteoblasts plus human triple negative MDA-MB-231 GFP/Luc2 breast cancer cells (control; Figure 5b) or (b) EO-231 cells plus human triple negative MDA-MB-231 GFP/Luc2 breast cancer cells (Figure 5c). Intratibial injection of human triple negative MDA-MB-231 GFP/Luc2 breast cancer cells alone served as an additional control (Figure 5a). The mice were sacrificed eight weeks post-injection, when their tibiae were harvested, sectioned, then stained for the presence of TRAP-positive osteoclasts (Figure 5a–c). Osteoclast surface (Oc.S) (Figure 5d) and osteoclast surface (Oc.S) per bone perimeter (B.Pm) (Figure 5e) were quantified per image for each condition. Oc.S and Oc.S/B.Pm are well-established unit used to quantify osteoclast formation in-vivo [71].

TRAP-positive, multinucleated (≥3 nuclei) osteoclasts were observed in each condition (Figure S4, arrows). Interestingly, we observed alterations in TRAP staining between bone sections of mice injected with MDA-MB-231 breast cancer cells plus naïve osteoblasts versus mice injected with MDA-MB-231 breast cancer cells plus EO-231 cells (Figure 5). Osteoclasts produced from an admix of MDA-MB-231 breast cancer cells plus naïve osteoblasts were elongated and more spread out (Figure 5b inset) compared to the smaller, more rounded osteoclasts produced from an admix of MDA-MB-231 breast cancer cells plus EO-231 cells (Figure 5c inset). After analyzing images from each condition, we found that the surface size of TRAP-positive osteoclasts produced in the presence of MDA-MB-231 breast cancer cells plus EO-231 cells was significantly reduced (57%) compared to the surface size of TRAP-positive osteoclasts produced in the presence of MDA-MB-231 breast cancer cells plus naïve osteoblasts (Figure 5d). When normalized to bone perimeter, we found a 62% reduction in the surface size of TRAP-positive osteoclasts produced in the presence of MDA-MB-231 breast cancer cells plus EO-231 cells when compared to the surface size of TRAP-positive osteoclasts produced in the presence of MDA-MB-231 breast cancer cells plus naïve osteoblasts (Figure 5e). This data further corroborates our in-vitro findings that osteoclasts produced in the presence of EO-231 cells have altered morphology and are smaller in size than osteoclasts produced in the presence of naïve osteoblasts.

As additional controls and to account for any potential alterations in soluble factors that may be produced by GFP-MDA-MB-231 cells versus parental MDA-MB-231 cells, we also tri-cultured RAW 264.7 pre-osteoclasts in the presence of exogenous RANKL for six days with MDA-MB-231 GFP/luc2 human triple negative breast cancer cells (i) plus naïve osteoblasts (control) or (ii) plus EO-231 cells in-vitro (Figure S5). Similar to data observed in Figure 2b–e, we observed an approximately 40% reduction in the number of TRAP-positive, multinucleated (≥3 nuclei) osteoclasts produced in the presence of EO-231 cells plus MDA-MB-231 GFP/luc2 breast cancer cells when compared to the number of TRAP-positive, multinucleated (≥3 nuclei) osteoclasts produced in the presence of naïve osteoblasts plus MDA-MB-231 GFP/luc2 breast cancer cells (Figure S5d).

### 3.5. DC-STAMP Expression Is Altered in Osteoclasts Exposed to EO CM

A critical step in the transformation of osteoclast precursors into mature, bone-resorbing osteoclasts is cell–cell fusion [72] (Figure 1B). In response to RANKL stimulation, single-nucleated osteoclast precursors fuse together to form large, multinucleated (≥3 nuclei) mature osteoclasts [33]. Dendrocyte expressed seven transmembrane protein (DC-STAMP) is an essential regulator of osteoclast fusion [32] and is used as a cell-fusion marker for osteoclast fusion in-vitro [33]. Since we observed osteoclasts that were smaller when produced in the presence of EO cells or exposed to EO CM, we next wanted to determine if osteoclast fusion was being altered.

RAW 264.7 pre-osteoclasts were exposed to naïve OB CM (control), EO-231 CM, or EO-MCF-7 CM in the presence of exogenous RANKL for three or six days. Three and six-day timepoints were used to determine differences in DC-STAMP expression between early-stage osteoclast fusion and late-stage osteoclast fusion, respectively. RAW 264.7 pre-osteoclasts maintained in osteoclast differentiation medium (vehicle medium; VM) in the presence of exogenous RANKL for three or six days were used as additional controls. After three or six days, protein lysates for each condition were collected and examined for alterations in DC-STAMP protein expression.

**Figure 6:**
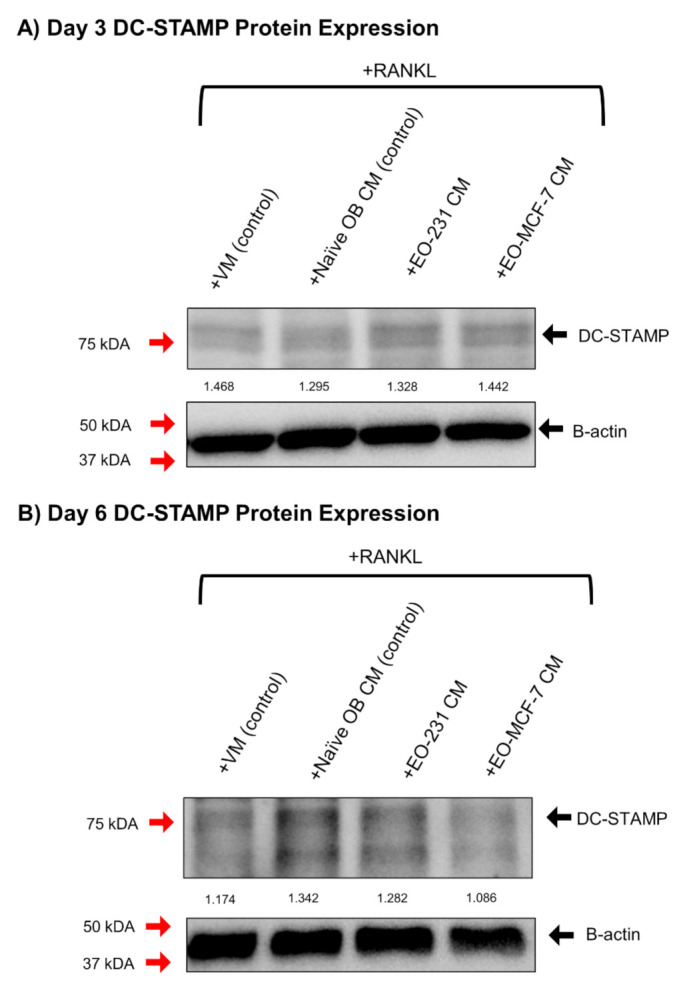
DC-STAMP Expression is Reduced in Pre-Osteoclasts Exposed to EO CM. RAW 264.7 pre-osteoclasts were exposed to naïve OB CM (control), EO-231 CM, or EO-MCF-7 CM in the presence of 50 ng/mL exogenous RANKL for (**A**) three days, or (**B**) six days. RAW 264.7 pre-osteoclasts maintained in vehicle media (VM) in the presence of 50 ng/mL exogenous RANKL for (**A**) three days, or (**B**) six days served as additional controls. Uncropped Western Blots are available in Figures S6 and S7. Cell lysates were collected and subjected to western blot for the osteoclast fusion factor, dendrocyte expressed seven transmembrane protein (DC-STAMP; black arrow). The red arrow indicates 75 kDa, 50 kDa, and 37 kDa, respectively. β-actin (black arrow) served as a loading control. Values shown are band densitometry measurements for DC-STAMP protein expression, which was normalized to β-actin protein expression. For day 3, DC-STAMP protein expression densitometry values, as normalized to β-actin protein expression, were 1.468 (+VM; control), 1.295 (+Naïve OB CM; control), 1.328 (+EO-231 CM), and 1.442 (+EO-MCF-7 CM). For day 6, DC-STAMP protein expression densitometry values, as normalized to β-actin protein expression, were 1.174 (+VM; control), 1.342 (+Naïve OB CM; control), 1.282 (+EO-231 CM), and 1.086 (+EO-MCF-7 CM).

We found that DC-STAMP protein expression was unchanged for osteoclasts differentiated to three days (Figure 6A and Figure S6). Densitometry measurements showed osteoclasts exposed to VM (control; 1.468), naïve OB CM (control; 1.295), EO-231 CM (1.328), or EO-MCF-7 CM (1.442) for three days had similar expression of DC-STAMP when normalized to β-actin (Figure 6A and Figure S6). This result was not surprising, considering mature osteoclasts are only just beginning to form by day 3 in-vitro [55].

We observed decreased DC-STAMP protein expression in osteoclasts exposed to either EO-231 CM or EO-MCF-7 CM for six days compared to osteoclasts exposed to naïve OB CM (control) for six days (Figure 6B and Figure S7). Densitometry measurements confirmed osteoclasts exposed to EO-231 CM (1.282) and EO-MCF-7 CM (1.086) had decreased DC-STAMP protein expression compared to osteoclasts exposed to naïve OB CM (control; 1.342) or VM control (1.174) (Figure 6B and Figure S7). We also observed differences in DC-STAMP expression when comparing osteoclasts produced in the presence of vehicle media compared to osteoclasts exposed to naïve OB CM (Figure 6B). The addition of exogenous RANKL to cultures containing naïve OB CM increases the concentration of soluble RANKL over and above that found in the vehicle media condition. Therefore, more soluble RANKL is expressed in cultures containing naïve OB CM compared to vehicle media, leading to increased osteoclast differentiation and subsequent fusion at day 6. These data suggest that osteoclasts produced in the presence of EO CM exhibit decreased osteoclast fusion, which further corroborates our findings of osteoclasts smaller in size when produced in the presence of EO CM.

### 3.6. EO Cells Have Altered Expression of Osteoclast-Associated Factors, TNFα and OPG

Previous work from our laboratory demonstrated that EO cells have altered expression of proteins compared to naïve osteoblasts [40]. Therefore, we wanted to determine if EOs have alterations in proteins involved in osteoclastogenesis, in particular: RANKL, OPG, and TNFα.

To initiate osteoclastogenesis, osteoblasts secrete M-CSF and soluble RANKL, the two factors needed for osteoclast differentiation [23] (Figure 1A). RANKL can exist in two forms on osteoblasts: a bound form and soluble form [22] (Figure 1A). Soluble RANKL will bind to the RANK receptor, and soluble M-CSF will bind the c-FMS receptor on osteoclast progenitor cells [23] (Figure 1A). The binding of these two factors stimulates downstream activation of genes associated with osteoclast differentiation, maturation, and activation [73,74]. To modulate osteoclast differentiation, OPG, secreted from osteoblasts, is a soluble protein known to bind soluble RANKL [24] (Figure 1A). In this way, OPG acts as a decoy receptor for RANKL, subsequently inhibiting RANKL from binding to the RANK receptor on osteoclast progenitors [34,75] (Figure 1A). 

Additionally, TNFα is a soluble protein found in the bone microenvironment and is known to initiate osteoclast differentiation independent of RANKL [28]. TNFα is secreted by many stromal cells found in the bone, including osteoblasts and macrophages [28,76] and when secreted, binds to the tumor necrosis factor receptor 1 (TNFR1) on osteoclast progenitor cells to initiate osteoclast differentiation [28,30] (Figure 1A).

**Figure 7:**
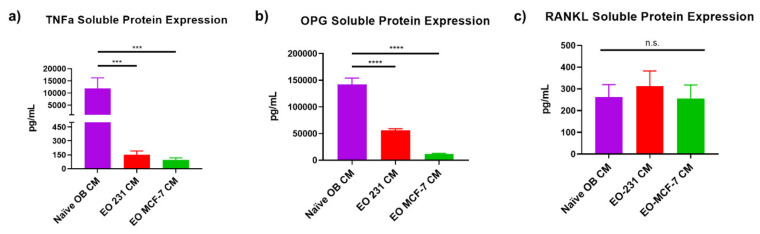
EOs exhibit decreased protein expression of osteoclast-associated factors TNFα and OPG. Naïve OB CM (control), EO-231 CM, and EO-MCF-7 CM were subjected to sandwich ELISA to assess alterations in the soluble protein production of (**a**) TNFα, (**b**) OPG, and (**c**) RANKL. Three individual batches of naïve OB CM, EO-231 CM, and EO-MCF-7 CM were assayed. *N* = 3. *** *p* < 0.001; **** *p* < 0.0001; n.s. = not significant.

To examine alterations in soluble protein expression of osteoclast-associated factors TNFα, OPG, and RANKL, conditioned medium was collected from naïve osteoblasts (control), EO-231 cells, or EO-MCF7 cells and subjected to sandwich ELISAs. TNFα soluble protein expression was reduced 77.5-fold in EO-231 CM and 122.5-fold in EO-MCF-7 CM when compared to naïve OB CM (Figure 7a). We also found that OPG soluble protein expression was reduced 2.5-fold in EO-231 CM and 12-fold in EO-MCF-7 CM when compared to naïve OB CM (Figure 7b). Interestingly, we found RANKL soluble protein expression to be unchanged between EO CM and naïve OB CM (Figure 7c). Thus, these data suggest that RANKL-independent mechanisms, such as TNFα, may be partially responsible for alterations in osteoclast formation when EO cells or their CM are present.

### 3.7. Soluble Protein TNFα Modulates Osteoclast Formation

We have shown that TNFα soluble protein expression was significantly reduced in EO CM compared to naïve OB CM (Figure 7a). TNFα has been shown to directly stimulate osteoclast formation independently of RANKL [28,77] and indirectly by stimulating osteoblasts to secrete RANKL [78,79]. Given this information, we next wanted to determine if the rescue of TNFα expression to expression levels seen in naïve OB CM would restore osteoclast formation in the presence of EO CM to that observed in controls (i.e., +RANKL or +Naïve OB CM).

**Figure 8:**
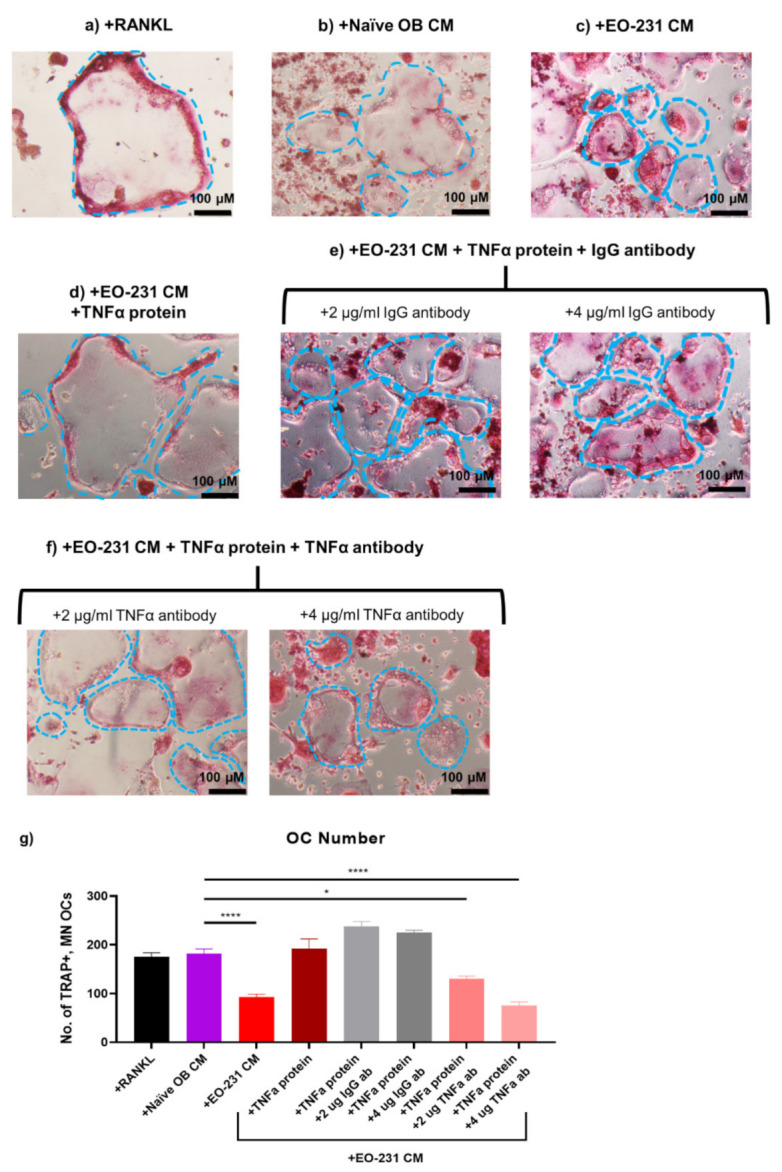
EO-231-altered Osteoclast Formation can be Modulated by TNFα. RAW 264.7 pre-osteoclasts were cultured in the presence of (**a**) 50 ng/mL exogenous RANKL, or cultured in the presence of 50 ng/mL exogenous RANKL and exposed for six days to (**b**) naïve OB CM (control) or (**c**) EO-231 CM. RAW 264.7 pre-osteoclasts cultured in the presence of 50 ng/mL exogenous RANKL and exposed to EO-231 CM plus (**d**) recombinant TNFα protein (12 ng/mL) or (**f**) recombinant TNFα protein (12 ng/mL) plus TNFα antibody (2 µg/mL or 4 µg/mL) for six days. (**e**) RAW 264.7 pre-osteoclasts were exposed to EO-231 CM in the presence of recombinant TNFα protein (12 ng/mL) plus polyclonal IgG antibody (2 µg/mL or 4 µg/mL) in the presence of 50 ng/mL exogenous RANKL for six days as a control. After six days, cultures were stained for tartrate resistant acid phosphatase (TRAP; pink color; blue outlines), a common marker for mature osteoclasts. The number (No.) of TRAP-positive, multinucleated (≥3 nuclei) mature osteoclasts were quantified for each condition (**g**). Scale = 100 µM. N = 3. * *p* < 0.05; **** *p* < 0.0001.

Since TNFα soluble protein expression was decreased in EO CM, we first exposed RAW 264.7 pre-osteoclasts to EO-231 CM plus TNFα recombinant protein in the presence of exogenous RANKL (Figure 8d). RAW 264.7 pre-osteoclasts maintained in osteoclast differentiation medium in the presence of exogenous RANKL (Figure 8a) or exposed to naïve OB CM in the presence of exogenous RANKL (Figure 8b) for six days served as controls. RAW 264.7 pre-osteoclasts were also exposed to EO-231 CM in the presence of exogenous RANKL for six days (Figure 8c). After six days, cultures were stained for TRAP (Figure 8a-d) and the number of TRAP-positive, multinucleated (≥3 nuclei) osteoclasts were quantified (Figure 8g). Addition of exogenous TNFα protein to EO-231 CM increased the number of TRAP-positive, multinucleated (≥3 nuclei) osteoclasts comparable to the number of TRAP-positive, multinucleated (≥3 nuclei) osteoclasts produced in the presence of naïve OB CM (Figure 8g).

Next, we assessed whether the neutralization of TNFα expression in the presence of EO-231 CM plus TNFα recombinant protein would return osteoclast formation to levels observed upon exposure to EO-231 CM alone (Figure 2e and Figure 8g). We exposed RAW 264.7 pre-osteoclasts to EO-231 CM plus TNFα recombinant protein plus TNFα neutralizing antibody in the presence of exogenous RANKL for six days (Figure 8f). As controls, RAW 264.7 pre-osteoclasts were exposed to EO-231 CM plus TNFα recombinant protein plus polyclonal IgG isotype antibody (Figure 8e). Some studies have suggested that osteoclastogenesis can be initiated by the binding of immune complexes (e.g., IgG) to Fcγ receptors on pre-osteoclasts [80]. To rule out this possibility, we incorporated an IgG isotype antibody as a negative control (Figure 8e).

As illustrated in Figure 8g, we observed a dose-response reduction in the number of osteoclasts formed in the presence of EO-231 CM plus addition of recombinant TNFα protein plus increasing amounts of TNFα neutralizing antibody. Modulation of levels of TNFα protein in the EO-231 CM permitted restoration of levels at or below that observed upon exposure to EO-231 CM alone (Figure 8g). Specifically, we observed a 28% reduction in the number of osteoclasts formed in the presence of EO-231 CM with the addition of 2 µg/mL TNFα neutralizing antibody plus TNFα recombinant protein. With the addition of 4 µg/mL TNFα neutralizing antibody, the reduction in osteoclast number was more robust, whereby we observed a 59% reduction in the number of osteoclasts formed in the presence of EO-231 CM with the addition of 4 µg/mL TNFα neutralizing antibody plus TNFα recombinant protein (Figure 8g). Furthermore, we found that the number of osteoclasts formed in the presence of EO-231 CM with the addition of polyclonal IgG antibody (+2 or +4 µg/mL) plus TNFα recombinant protein were comparable to the number of osteoclasts formed in the presence of EO-231 CM plus TNFα recombinant protein (Figure 8g), suggesting that the addition of polyclonal IgG antibody did not independently affect osteoclast formation.

**Figure 9:**
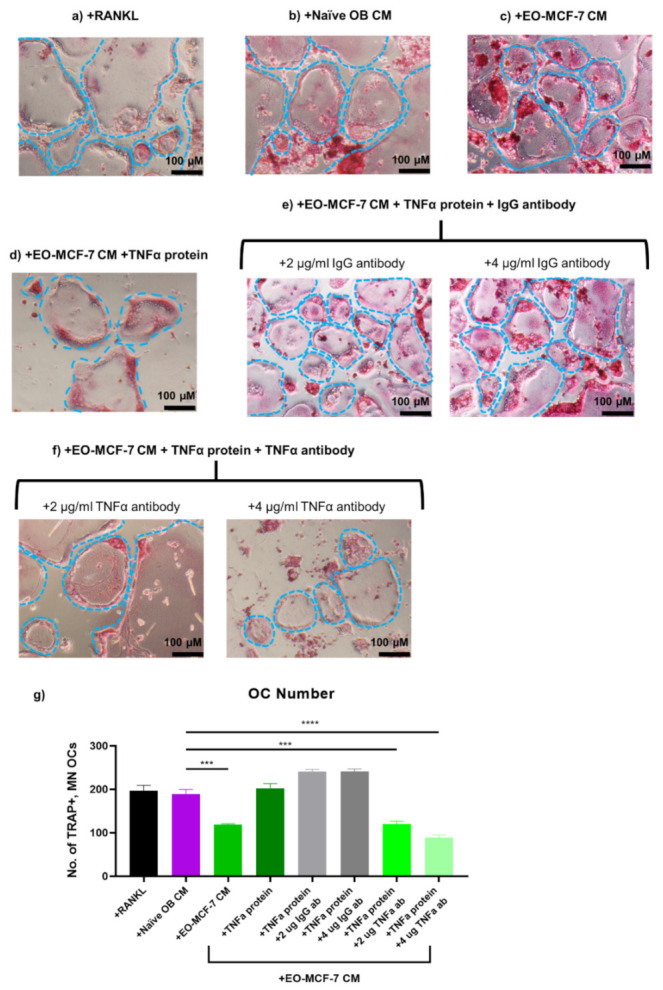
EO-MCF-7-altered Osteoclast Formation can be Modulated by TNFα. RAW 264.7 pre-osteoclasts were cultured in the presence of (**a**) 50 ng/mL exogenous RANKL (**a**) or cultured in the presence of 50 ng/mL exogenous RANKL and exposed either to (**b**) naïve OB CM (control) or (**c**) EO-MCF-7 CM for six days. RAW 264.7 pre-osteoclasts were cultured in the presence of 50 ng/mL exogenous RANKL for six days, then exposed to EO-MCF-7 CM plus either (**d**) recombinant TNFα protein (12 ng/mL) or (**f**) recombinant TNFα protein (12 ng/mL) plus TNFα antibody (2 µg/mL or 4 µg/mL). (**e**) RAW 264.7 pre-osteoclasts were exposed to EO-MCF-7 CM in the presence of recombinant TNFα protein (12 ng/mL) plus polyclonal IgG antibody (2 µg/mL or 4 µg/mL) in the presence of 50 ng/mL exogenous RANKL for six days as a control. After six days, cultures were stained for tartrate resistant acid phosphatase (TRAP; pink color; blue outlines), a common marker for mature osteoclasts. The number (No.) of TRAP-positive, multinucleated (≥3 nuclei) mature osteoclasts were quantified for each condition (**g**). Scale = 100 µM. *N* = 3. *** *p* < 0.001; **** *p* < 0.0001.

We also exposed RAW 264.7 pre-osteoclasts in the presence of exogenous RANKL to EO-MCF-7 CM plus TNFα recombinant protein (Figure 9d). RAW 264.7 pre-osteoclasts in the presence of exogenous RANKL and maintained in osteoclast differentiation medium (Figure 9a) or exposed to naïve OB CM in the presence of exogenous RANKL (Figure 9b) for six days served as controls. RAW 264.7 pre-osteoclasts were also exposed to EO-MCF-7 CM in the presence of exogenous RANKL (Figure 9c). After six days, cultures were stained for TRAP (Figure 9a–d) and the number of TRAP-positive, multinucleated (≥3 nuclei) osteoclasts were quantified (Figure 9g). Addition of exogenous TNFα protein to EO-MCF-7 CM increased the number of TRAP-positive, multinucleated (≥3 nuclei) osteoclasts comparable to the number of TRAP-positive, multinucleated (≥3 nuclei) osteoclasts produced in the presence of naïve OB CM (Figure 9g).

We next assessed whether the neutralization of TNFα expression in the presence of EO-MCF-7 CM plus TNFα recombinant protein would return osteoclast formation to levels observed upon exposure to EO-MCF-7 CM alone (Figure 2e and Figure 8g). We exposed RAW 264.7 pre-osteoclasts to EO-MCF-7 CM plus TNFα recombinant protein plus TNFα neutralizing antibody in the presence of exogenous RANKL for six days (Figure 9f). As controls, RAW 264.7 pre-osteoclasts were exposed to EO-MCF-7 CM plus TNFα recombinant protein plus polyclonal IgG isotype antibody (Figure 9e). Modulation of levels of TNFα protein in the EO-MCF-7 CM permitted restoration of levels at or below that observed upon exposure to EO-MCF-7 CM alone (Figure 9g). Specifically, we observed a 36% reduction in the number of osteoclasts formed in the presence of EO-MCF-7 CM with the addition of 2 µg/mL TNFα neutralizing antibody plus TNFα recombinant protein and a 54% reduction in the number of osteoclasts formed in the presence of EO-MCF-7 CM with the addition of 4 µg/mL TNFα neutralizing antibody plus TNFα recombinant protein (Figure 9g). Moreover, we found that the number of osteoclasts formed in the presence of EO-MCF-7 CM with the addition of polyclonal IgG antibody (+2 or +4 µg/mL) plus TNFα recombinant protein were comparable to the number of osteoclasts formed in the presence of EO-MCF-7 CM plus TNFα recombinant protein (Figure 9g), suggesting that the addition of polyclonal IgG antibody did not independently affect osteoclast formation.

We also utilized CD11b+ primary bone marrow monocytes (BMMs) to further assess the effects of TNFα on osteoclast maturation in-vitro. CD11b+ primary BMMs in the presence of exogenous M-CSF and exogenous RANKL were exposed for six days to (a) naïve OB CM (control), (b) EO-231 CM plus TNFα recombinant protein, and (c) EO-MCF-7 CM *plus* TNFα recombinant protein (Figure S8a–c). We found that the addition of TNFα recombinant protein to EO CM increased the number of TRAP-positive, multinucleated (≥3 nuclei) osteoclasts produced in the presence of naïve OB CM (Figure S8f). We also assessed whether the neutralization of TNFα expression in the EO CM plus TNFα recombinant protein would reduce osteoclast formation. CD11b+ primary BMMs in the presence of exogenous M-CSF and exogenous RANKL were exposed for six days to EO CM plus TNFα recombinant protein plus TNFα neutralizing antibody (Figure S8d–e). Modulation of levels of TNFα protein plus TNFα neutralizing antibody reduced osteoclast formation in-vitro similar to levels seen with RAW 264.7 pre-osteoclasts exposed to EO CM plus TNFα recombinant protein plus TNFα neutralizing antibody (Figure S8f,g and Figure 9g). Thus, these data suggest that alterations in osteoclast formation by EOs are mediated, in part, by TNFα.

## 4. Discussion

Osteoclast formation occurs in two different ways: (1) either by direct cell-to-cell contact or (2) via soluble factor crosstalk. In situations of direct cellular communication, osteoblasts express RANKL on their surface, which binds to the RANK receptor on pre-osteoclast cells, initiating osteoclastogenesis via pathways including PI3K, NF-KB, and MAPK [81]. In addition to the RANKL–RANK signaling axis, it was recently discovered that leucine-rich repeat-containing G-protein-coupled receptor 4 (LGR4) also serves as a receptor on osteoclasts for RANKL [82]. Luo and colleagues showed that LGR4 physically interacts with RANKL, activating G-protein signaling in osteoclast precursors via Gα_q._ Interestingly, LGR4 was found to compete with the RANK receptor for binding of RANKL, opposing pro-osteoclast actions, and also negatively regulated the nuclear translocation of NFATC1, a key transcription factor in osteoclastogenesis, via expression of Gα_q_ [82].

In addition to direct cellular contact, osteoclastogenesis also occurs via soluble factor crosstalk. Osteoblasts additionally secrete soluble RANKL, which can bind the RANK receptor on osteoclast precursors. As a regulator of this process, osteoblasts produce osteoprotegerin (OPG). OPG is a decoy receptor for RANKL. The ratio of OPG: RANKL helps control bone resorption through regulation of osteoclastogenesis. In situations when osteoclastogenesis needs to be suppressed, increased expression of OPG will lead to increased OPG binding with RANKL. In situations when osteoclastogenesis needs to be increased, less OPG will be produced, allowing RANKL to bind with the RANK receptor. Osteoclastogenesis can also be stimulated by RANKL independent mechanisms including TNFα, IL-6, and TGF-β [29,31]. TNFα can bind to the TNF receptor on pre-osteoclasts and initiate osteoclastogenesis via IKK signaling and NF-KB. Moreover, soluble IL-6 is capable of binding to the gp130 receptor on the membrane of pre-osteoclasts and initiating osteoclast formation via JAK/STAT signaling [31]. TGF-β-induced osteoclast formation has been shown to independently activate downstream NF-ΚB signaling and initiate osteoclast differentiation [29,83]. In the study described here, we specifically focused on osteoclast formation via RANKL and TNFα signaling.

During normal bone remodeling, osteoblasts and osteoclasts communicate with one another to remodel bone [26]. Osteoblasts secrete factors, such as RANKL, that stimulate osteoclast progenitors to become mature osteoclasts [10]. However, when breast cancer cells enter the bone, communication between osteoclasts and osteoblasts is disrupted, altering bone remodeling and leading to increased bone destruction [11]. Breast cancer cells overstimulate osteoblast secretion of RANKL, causing sustained osteoclast formation and maturation [7]. In this way, osteoclasts become overactive and continually resorb bone, while osteoblasts fall short in building new bone [35]. Sustained osteoclast resorption releases sequestered cytokines and growth factors stored within the bone matrix, which breast cancer cells use to promote cancer growth [39,65]. Our laboratory has previously identified a novel subpopulation of osteoblasts found in the bone-tumor niche, termed “educated” osteoblasts (EOs) [40]. The focus of this study was to understand how osteoclastogenesis is affected by EO cells.

To determine how osteoclastogenesis is affected by EO cells, we first wanted to determine whether soluble factors from EO CM were capable of altering osteoclast formation in-vitro. We observed a 65% reduction in the number of TRAP-positive, multinucleated (≥3 nuclei) osteoclasts exposed to EO-231 CM and a 52% reduction in the number of TRAP-positive, multinucleated (≥3 nuclei) osteoclasts exposed to EO-MCF-7 CM when compared to osteoclasts exposed to naïve OB CM (Figure 2e). Additionally, we observed that osteoclasts exposed to EO-231 CM were 71% smaller and osteoclasts exposed to EO-MCF-7 CM were 72% smaller than osteoclasts exposed to naïve OB CM (Figure 2f). This was a surprising observation and could suggest that EO cells reduce osteoclastogenesis and delay progression to the vicious cycle of bone degradation. Importantly, these data demonstrate that osteoclast formation is altered by soluble factors secreted by EO cells.

As a result of this observation, we next wanted to determine how osteoclast formation was modulated by direct cellular contact. We found a 25% reduction in the number of osteoclasts formed in the presence of EO-231 cells and a 19% reduction in the number of osteoclasts formed in the presence of EO-MCF-7 cells compared to osteoclasts produced in the presence of naïve osteoblasts (Figure 2j). Additionally, we observed that osteoclasts produced in the presence of EO-231 cells or EO-MCF-7 cells were 33% smaller and 12% smaller, respectively, compared to osteoclasts produced in the presence of naïve osteoblasts (Figure 2k). These data suggest that pre-osteoclast direct cell contact with EO cells produces osteoclasts that are less in number and smaller in size, further suggesting that EO cells reduce osteoclast formation, which may delay progression to the vicious cycle of breast cancer bone metastasis. Overall, these results suggest that both soluble factors found in EO CM and direct contact with EO cells decrease osteoclast formation.

CD11b+ primary bone marrow monocytes were utilized as another model to assess the affects EO cells have on osteoclast maturation. Although studies have shown that RAW 264.7 cells and CD11b+ primary bone marrow monocytes closely resemble one another in phenotype and function [62,84], there have been reports about inconsistencies between RAW 264.7 pre-osteoclasts and primary bone marrow monocytes [85,86,87]. One group found that there are inconsistencies in macrophage marker expression. In particular, F4/80, a macrophage marker, was highly expressed in primary bone marrow macrophages compared to RAW cells, which expressed lower levels of F4/80 [84], while another group found that F4/80 was unchanged between RAW 264.7 cells and primary bone marrow macrophages [62]. In addition, RAW 264.7 cells are a macrophage-monocyte cell line derived from the lymphoma of a male BALB/c mouse infected by the Abelson murine leukemia virus (A-muLV), resulting in a constitutive activation of v-Abl [44]. This type of virus causes constitutive activations of signaling cascades involved in proliferation, including Ras, Jak-stat, JNK, Erk, and PI3K/Akt [58,88]. Ng and colleagues further identified that Erk and Akt were constitutively active due to constitutive activation of v-Abl mutation [85]. Conversely, CD11b+ cells are isolated directly from murine bone marrow and are therefore not transformed in any way. CD11b+ primary bone marrow monocytes have been shown, upon stimulation with exogenous M-CSF [89] and exogenous RANKL, to differentiate into osteoclasts in-vitro [59,90]. In addition, RAW 264.7 cells can differentiate without the presence of exogenous M-CSF in-vitro [45,46,47], whereas CD11b+ cells cultured in-vitro need M-CSF to survive [89]. Thus, to alleviate any potential discrepancies between pre-osteoclasts used, we also confirmed our results using primary bone marrow monocytes. We observed a 58% reduction in the number of osteoclasts produced from a co-culture of CD11b+ primary bone marrow monocytes and EO-231 cells (Figure S2d). Additionally, we observed a 50% reduction in the number of osteoclasts produced from a co-culture of CD11b+ primary bone marrow monocytes and EO-MCF-7 cells (Figure S2d). These data confirm our prior results using RAW 264.7 cells and further suggest that osteoclasts produced in the presence of EO cells are less in number (Figure 2).

We next wanted to determine how the alterations in osteoclast formation might be affecting osteoclast function. It is well known that mature, active osteoclasts form resorptive pits at sites of bone remodeling [23,68,70]. During bone remodeling, osteoclasts bind to the bone matrix and create a sealed area where enzymes are secreted to degrade bone [23,68]. This area of bone degradation by osteoclasts is referred to as a resorptive pit [56,69] and is used as a characterization of osteoclast function in-vitro [67]. Osteoclasts produced in the presence of EO cells were less in number and smaller in size (Figure 2 and Figure S2); thus, we hypothesized that osteoclasts produced in the presence of EO cells would also have decreased resorptive activity. To determine osteoclast function in-vitro, we co-cultured RAW 264.7 pre-osteoclasts with naïve osteoblasts (control) or EO cells on a bone-mimetic matrix in the presence of RANKL. Cultures were first stained for TRAP and imaged via light microscopy (Figure 3a–d), and then the cells were removed from the bone-like matrix to observe each osteoclast’s resorptive pit. To our knowledge, this is the first time simultaneous analysis of TRAP stain plus resorptive pit formation analysis of on osteoclasts on a bone-like matrix has been reported. We observed a 34% reduction in the number of TRAP-positive, multinucleated (≥3 nuclei) osteoclasts produced in the presence of EO-231 cells and a 13% reduction in the number of TRAP-positive, multinucleated (≥3 nuclei) osteoclasts produced in the presence of EO-MCF-7 cells compared to compared to TRAP-positive, multinucleated (≥3 nuclei) osteoclasts produced in the presence of naïve osteoblasts (Figure 3e). Furthermore, we observed osteoclasts produced in the presence of EO-231 cells resorbed 49% less bone-mimetic matrix, and osteoclasts produced in the presence of EO-MCF-7 cells resorbed 58% less bone-mimetic matrix compared to osteoclasts resorbing bone that were produced in the presence of naïve osteoblasts (Figure 3f). Importantly, we found that TRAP-positive, multinucleated (≥3 nuclei) osteoclasts produced in the presence of EO cells have approximately 50% less nuclei (~7–9 nuclei per cell) compared to TRAP-positive, multinucleated (≥3 nuclei) osteoclasts produced in the presence of naïve osteoblasts (~18–23 nuclei per cell) or exogenous RANKL (~16–19 nuclei per cell) (Figure 3h). Interestingly, in 1992, Piper et al. examined the relationship between the number of osteoclast nuclei and their resorptive capability in-vitro. The authors found that the greater the number of nuclei per osteoclast, the larger the volume of pit that was made, indicating an increased osteoclast resorptive capability and aggressiveness [91]. Our findings are in agreement with these data, suggesting that osteoclasts produced in the presence of EO cells resorb less bone and are less aggressive due to a smaller number of nuclei per cell when compared to osteoclasts produced in the presence of naïve osteoblasts or exogenous RANKL, which yield osteoclasts with double the number of nuclei per cell (Figure 3h). Coupled with our observation that osteoclasts produced in the presence of EO cells or their conditioned medium are less in number and smaller in size (Figure 2, Figure S2), these data further suggest that osteoclasts produced in the presence of EO cells also have reduced resorptive capabilities on a bone-like matrix. These data provide further evidence that, on a bone-like surface, osteoclastogenesis and bone resorption is reduced in the presence of EO cells.

Additionally, we found that osteoclasts produced on a bone mimetic matrix and in the presence of EO-231 cells were 18% smaller and osteoclasts produced in the presence of EO-MCF-7 cells were 28% smaller compared to osteoclasts produced on a bone mimetic matrix and in the presence of naïve osteoblasts (Figure 3g). These data support our prior in-vitro results (Figure 2) and further provide evidence that osteoclastogenesis is reduced by EO cells.

During metastatic progression in bone, communication between bone-forming osteoblasts and bone-resorbing osteoclasts is disrupted, resulting in increased osteoclast resorption of bone [11,92]. Tumor factors, including parathyroid hormone related protein (PTHrP), increase the secretion of RANKL from osteoblasts [35]. RANKL then binds to the RANK receptor on osteoclast progenitor cells increases osteoclast formation and subsequent activation of mature osteoclasts [35]. Mature osteoclasts become overactive and resorb bone at a higher rate than osteoblasts forming new bone, resulting in osteolytic lesion formation as seen in late-stage disease bone metastatic breast cancer [11,35,38]. Sustained bone degradation by osteoclasts increases the release of sequestered cytokines, growth factors, and minerals in the bone matrix, which breast cancer cells utilize to continue this cycle [35,37]. This “vicious cycle” of bone degradation is an important way breast cancer cells utilize osteoclasts and bone resorption to support tumor progression in bone [7,11]. Therefore, it was important to determine how breast cancer cells affect osteoclastogenesis when EO cells are present. To denote human disease, we chose two breast cancer cell lines: MDA-MB-231 human triple negative breast cancer cells and MCF-7 human ER+ breast cancer cells. We specifically picked these two subtypes of breast cancer based on their tropism for bone metastases. Triple negative breast cancer is considered a highly aggressive subtype of breast cancer, because it grows quickly, rapidly spreads to other organs, including bone, and is associated with high chemotherapy resistance [93,94]. Conversely, ER+ breast cancer has been shown to remain in a growth suppressive state in the skeleton for up to three decades in individuals with this breast cancer subtype [95,96]. To determine osteoclast formation, RAW 264.7 pre-osteoclasts were tri-cultured with breast cancer cells plus EO cells (Figure 4c) or plus naïve osteoblasts (control; Figure 4b) in the presence of exogenous RANKL for six days. As an additional control, RAW 264.7 pre-osteoclasts were co-cultured with breast cancer cells in the presence of exogenous RANKL for six days (Figure 4a). Cultures containing MDA-MB-231 breast cancer cells or MCF-7 breast cancer cells were TRAP stained to identify TRAP-positive, multinucleated (≥3 nuclei) osteoclasts (Figure 4b–d and f–h, respectively). We observed a 19% reduction in the number of TRAP-positive, multinucleated (≥3 nuclei) osteoclasts produced in the presence of MDA-MB-231 breast cancer cells plus EO-231 cells compared to TRAP-positive, multinucleated (≥3 nuclei) osteoclasts produced in the presence of MDA-MB-231 breast cancer cells plus naïve osteoblasts (Figure 4e). These experiments were repeated using human MDA-MB-231 GFP/luc2 breast cancer cells (Figure S5), whereby we observed a 40% reduction in the number of TRAP-positive, multinucleated (≥3 nuclei) osteoclasts produced in the presence of EO-231 cells plus MDA-MB-231 GFP/luc2 breast cancer cells (Figure S5d). This was in comparison to the number of TRAP-positive, multinucleated (≥3 nuclei) osteoclasts produced in the presence of naïve osteoblasts plus MDA-MB-231 GFP/luc2 breast cancer cells (Figure S5d). Similarly, we observed a 36% reduction in the number of TRAP-positive, multinucleated (≥3 nuclei) osteoclasts produced in the presence of MCF-7 breast cancer cells plus EO-MCF-7 cells compared to TRAP-positive, multinucleated (≥3 nuclei) osteoclasts produced in the presence of MCF-7 breast cancer cells plus naïve osteoblasts (Figure 4i). Overall, the number of TRAP-positive, multinucleated (>3 nuclei) osteoclasts produced from a tri-culture of pre-osteoclasts plus MDA-MB-231 breast cancer cells plus EO-231 cells or a tri-culture of pre-osteoclasts plus MCF-7 breast cancer cells plus EO-MCF-7 breast cancer cells were reduced compared to the number of osteoclasts formed from tri-cultures of pre-osteoclasts plus MDA-MB-231 breast cancer cells plus naïve osteoblasts or tri-culture of pre-osteoclasts plus MCF-7 breast cancer cells plus naïve osteoblasts (Figure 4e,i).

Of particular note, while our data have demonstrated that osteoclastogenesis and bone resorption is reduced in the presence of EO cells or their conditioned media, we additionally observed statistically significant differences between osteoclasts generated in the presence of EO-MCF-7 cells or ER+ MCF-7 cells plus EO-MCF-7 cells as opposed to osteoclasts generated in the presence of EO-231 cells or triple negative MDA-MB-231 cells plus EO-231 cells. Importantly, we noted that osteoclasts produced on a bone mimetic surface in the presence of EO-MCF-7 cells resorb 58% less matrix than control (Figure 3f), compared to osteoclasts produced on a bone mimetic surface in the presence of EO-231 cells, which resorb 49% less matrix than control (Figure 3e), a nearly 10% difference in osteoclast resorptive capability. Under the same conditions, we also noticed a 10% difference in osteoclast size, where osteoclasts formed in the presence of EO-MCF-7 cells on a bone mimetic surface were 10% smaller than those formed on a bone mimetic surface in the presence of EO-231 cells (Figure 3g). Furthermore, when breast cancer cells were added to co-cultures, we observed a two-fold difference in the reduction in the number of osteoclasts formed in the presence of EO-MCF-7 cells plus MCF-7 cells (36% reduction, Figure 4i), compared to osteoclasts formed in the presence of EO-231 cells plus MDA-MB-231 cells (19% reduction, Figure 4e).

Previous studies have demonstrated that ER+ breast cancer cells can lie dormant, remaining in a non-proliferative state in the skeleton for over two decades [95,96]. Importantly, our data demonstrates that EO cells reduce osteoclastogenesis, specifically, we observed a robust decrease in osteoclastogenesis when ER+ MCF-7 breast cancer cells plus EO-MCF-7 cells were present (Figure 4i). Furthermore, we observed a significant reduction in the amount of matrix resorbed (58% less matrix resorbed, Figure 3f) when osteoclasts were formed on a bone mimetic surface in the presence of EO-MCF-7 cells. It should be noted, however, that a reduction in the amount of matrix resorbed by osteoclasts produced in the presence of EO cells may also be due to less osteoclasts being present (i.e., Figure 2e,j) when compared to osteoclasts formed in the presence of naïve osteoblasts. To attempt to answer this question, in Table S1, we estimated the bone resorptive activity per individual osteoclast for each condition by dividing the average resorbed area (Figure 3f) by the average number of osteoclasts per area (Figure 3e). The results in Supplementary Table S1 demonstrate that an individual TRAP-positive, multinucleated (≥3 nuclei) osteoclast produced in the presence of EO-231 cells or EO-MCF-7 cells resorbs less bone-like matrix compared to an individual osteoclast produced in the presence of naïve osteoblasts (approximately 30% less bone-like matrix) or an individual osteoclast produced in the presence of exogenous RANKL (approximately three-fold less bone-like matrix). Thus, these data imply that EO cells reduce osteoclast resorptive capabilities.

Importantly, our data suggest that EO cell reduction of osteoclast activation is especially relevant in earlier stages of breast cancer dissemination to the skeleton, when outgrowth of macrometastatic lesions and advanced osteolysis has not yet occurred. Expanding on our prior data, which suggested that EO cell activity is increased in early-stage disease [40], it may be the case that reduced osteoclast activity is a direct consequence of increased tumor-inhibitory activity of EO cells. An alternative explanation is that reduced osteoclast activity may in fact promote a dormant tumor microenvironment, given evidence that osteoclast activation specifically has been shown in the literature to promote dormant tumor cell re-awakening [42]. A combination of these two events may also occur. Regardless, these data suggest there may be a therapeutic window in early-stage disease to capitalize on both the tumor-inhibitory effects of EO cells and comparatively reduced activity of osteoclasts. Indeed, as events that are currently unknown awaken proliferatively quiescent bone disseminated tumor cells, macrometastatic lesion formation is accompanied with overactivation of osteoclasts in late-stage disease. Thus, the tumor-inhibitory functions of EO cells may, in part, help regulate the balance between a tumor-suppressive and tumor-promoting niche. On-going work in our laboratory is aimed at directly elucidating these events as potential therapeutic targets [7,11,35]

To replicate our in-vitro results in-vivo, we used an intratibial model of bone metastasis to study how osteoclastogenesis is affected by EO cells. To assess for differences in osteoclast formation in-vivo, an admix of naïve osteoblasts plus MDA-MB-231 breast cancer cells (control) or an admix of MDA-MB-231 breast cancer cells plus EO-231 cells were injected into the tibia of mice. MDA-MB-231 breast cancer cells injected alone were used as an additional control. Similar to our in-vitro results, we found that osteoclasts produced in bone upon injection of an admix of MDA-MB-231 triple negative breast cancer cells plus EO-231 cells were about 50% smaller in size compared to osteoclasts produced in bone upon an injection an admix of MDA-MB-231 triple negative breast cancer cells plus naïve osteoblasts or MDA-MB-231 triple negative breast cancer cells alone (Figure 5d–e). Thus, these data suggest that the increased presence of EO cells in the bone microenvironment reduces osteoclast formation in-vivo.

Osteoclast fusion is a critical step for immature osteoclasts to become mature, functional osteoclasts [33,97] (Figure 1B). Dendrocyte expressed seven transmembrane protein (DC-STAMP) is a master regulator of osteoclast fusion [33]. DC-STAMP is a receptor found on osteoclasts and as osteoclast fusion occurs, DC-STAMP expression increases [98]. Our data suggest that osteoclast formation is decreased in the presence of EO cells or their conditioned medium (Figure 2, Figure 3 and Figure 4 and Figure S2). To examine how osteoclast fusion is affected by EO cells, we exposed RAW 264.7 pre-osteoclasts to naïve osteoblast conditioned medium (control) or EO conditioned medium for three or six days. Since formation of mature osteoclasts is known to occur between 5–7 days in-vitro [55], we chose an early timepoint (day 3) and a later timepoint (day 6) to analyze DC-STAMP protein expression. At the early timepoint, we found no change in DC-STAMP protein expression between osteoclasts exposed to EO conditioned medium and osteoclasts exposed to naïve osteoblast conditioned medium (Figure 6A). This result was not surprising considering mature osteoclast formation starts to begin around day 3 in-vitro [55]. When analyzing the day 6 timepoint, we found that osteoclasts exposed to EO conditioned medium had decreased DC-STAMP protein expression compared to osteoclasts exposed to naïve osteoblast conditioned medium (Figure 6B). Our interpretation of these data demonstrate that pre-osteoclasts exposed to EO conditioned medium have decreased expression of osteoclast fusion factor DC-STAMP in the later stages of osteoclast maturation. To further support these results, we quantified the number of nuclei per TRAP-positive, multinucleated (≥3 nuclei) osteoclast in-vitro, and found that, on average, multinucleated (≥3 nuclei) osteoclasts formed in the presence of EO cells have approximately half as many nuclei as those formed either in the presence of naïve osteoblasts or exogenous RANKL (Figure 3h). This further supports our data showing dysregulated osteoclast fusion via a reduction in the expression of DC-STAMP in the presence of EO cells.

It could also be the case that decreased DC-STAMP expression is due to osteoclast apoptosis. Akchurin et al. have demonstrated that RAW 264.7 pre-osteoclasts cultured with exogenous RANKL for long periods of time (i.e., 15 days) go through periods of synchronized osteoclast differentiation and death [99]. The authors found that over a 15-day time period, RAW 264.7 osteoclasts went through two or three waves of osteoclast differentiation and death, with the first wave of osteoclast death starting at day 6 [99]. Conversely, Tanaka et al. have demonstrated that RANKL is an anti-apoptotic factor and that the RANKL pathway is pro-survival for osteoclasts [100]. Additionally, our laboratory and others have shown that osteoclast formation using RAW 264.7 pre-osteoclasts treated with RANKL occurs between days 3–7, with optimal osteoclast formation occurring between days 5–6 [55]. Our data demonstrate that osteoclast maturation is decreased when EO cells or their conditioned media is present (Figure 2). Furthermore, we also have shown that more than 75% of osteoclasts formed in our experiments are multinucleated (≥3 nuclei, Figure S1), suggesting that osteoclast fusion is indeed occurring. Therefore, we believe that for our system, decreased DC-STAMP protein expression is a result of decreased osteoclast maturation and fusion, and not osteoclast apoptosis.

Our data, in part, suggest that in addition to direct cell-to-cell contact, soluble factors secreted by EO cells also alter osteoclastogenesis by reducing osteoclast formation (Figure 2). To assess alterations in soluble factors secreted by EO cells that may alter osteoclastogenesis, we next analyzed the soluble protein expression of osteoclast-associated factors RANKL, OPG, and TNFα. RANKL, bound to or secreted from osteoblasts, is an important factor for osteoclast differentiation [97]. RANKL binds to the RANK receptor on osteoclast progenitor cells to initiate osteoclast differentiation [101] (Figure 1A). OPG, also secreted from osteoblasts, is a decoy receptor for RANKL, where it binds RANKL and inhibits it from binding to the RANK receptor on osteoclast progenitor cells [24] (Figure 1B). TNFα, which can be secreted from osteoblasts or macrophages found in the bone microenvironment, is a factor that can activate osteoclastogenesis independently of RANKL [28,102]. We subjected naïve osteoblast conditioned medium (control) and EO conditioned medium to sandwich ELISAs to determine the soluble protein production of RANKL, OPG, and TNFα. We observed a 77.5-fold reduction in TNFα soluble protein expression in EO-231 conditioned medium and a 122.5-fold reduction in TNFα soluble protein expression in EO-MCF-7 conditioned medium (Figure 7a). Additionally, we observed a 2.5-fold reduction in OPG soluble protein expression in EO-231 conditioned medium and a 12-fold reduction in OPG soluble protein expression in EO-MCF-7 conditioned medium (Figure 7b). Interestingly, we found no changes in RANKL soluble protein expression in EO conditioned medium compared to naïve osteoblast conditioned medium (Figure 7c). These data demonstrate that EOs exhibit decreased soluble protein expression of osteoclast-associated factors TNFα and OPG.

Furthermore, we were surprised to find in our system that soluble RANKL protein expression in naïve osteoblast and EO conditioned medium was low in comparison to amounts used in our positive controls: ~250–300 pg/mL RANKL in naïve osteoblast and EO conditioned medium (Figure 7c) when compared to 50 ng/mL exogenous RANKL used for positive controls (Figure 2). While we did find that exposure to naïve osteoblast or EO conditioned medium elicited the formation of TRAP-positive, multinucleated (≥3 nuclei) osteoclasts (~50–200 multinucleated osteoclasts; Figure 2b–e), this was in stark comparison to the number of multinucleated (≥3 nuclei) osteoclasts formed (~250) upon exposure to 50 ng/mL exogenous RANKL (positive control, Figure 2a,e). Even though our data show that the decoy receptor for soluble RANKL, OPG, is decreased in EO conditioned medium compared to naïve osteoblast conditioned medium, and given that comparatively low expression of soluble RANKL was present in the conditioned medium, we reasoned that other mechanisms beyond the ratio of soluble RANKL:OPG were regulating osteoclastogenesis when EO cells were present.

We further investigated TNFα as a potential mediator for decreased osteoclastogenesis. It has been reported in the literature that TNFα can directly stimulate osteoclast formation independently of RANKL [28,77]. Therefore, TNFα may be responsible for alterations in osteoclast formation by EO cells or their conditioned medium. Since TNFα expression was robustly reduced in EO conditioned medium, TNFα recombinant protein was added to cultures containing pre-osteoclasts exposed to EO conditioned medium in the presence of exogenous RANKL. We found that the addition of TNFα recombinant protein to cultures containing pre-osteoclasts exposed to EO conditioned medium increased the number of TRAP-positive, multinucleated (≥3 nuclei) osteoclasts to levels at or below that observed upon exposure to naïve osteoblast conditioned medium (Figure 8g and Figure 9g). 

Next, we neutralized recombinant TNFα protein with the addition of a TNFα antibody to restore osteoclast formation (Figure 8f–g and Figure 9f–g) to levels seen previously with the addition of EO conditioned medium alone (Figure 2e, Figure 8g and Figure 9g). We observed a dose-response reduction in the number of osteoclasts formed in the presence of EO conditioned medium with the addition of recombinant TNFα protein plus the addition of increasing concentrations of a TNFα antibody (Figure 8g and Figure 9g). Upon the addition of 2 µg/mL TNFα neutralizing antibody plus TNFα recombinant protein, we observed a 28% reduction in the number of osteoclasts formed in the presence of EO-231 conditioned medium and a 36% reduction in the number of osteoclasts formed in the presence of EO-MCF-7 conditioned medium (Figure 8g and Figure 9g). Furthermore, upon the addition of 4 µg/mL TNFα neutralizing antibody plus TNFα recombinant protein, this reduction was increased such that we observed a 59% reduction in the number of osteoclasts formed in the presence of EO-231 conditioned medium and a 54% reduction in the number of osteoclasts formed in the presence of EO-MCF-7 conditioned medium (Figure 8g and Figure 9g). We also recapitulated these experiments with CD11b+ primary bone marrow monocytes and found a similar trend comparable to experiments performed using RAW 264.7 pre-osteoclasts (Figure S8). These data suggest that TNFα is able to, in part, modify osteoclast formation as driven by EO cells.

Interestingly, anti-TNFα therapies are broadly used in the clinic and have been used as prognostic markers for patients with bone metastatic breast cancer [103]. It has been demonstrated that TNFα could be used as an independent prognostic marker for both progression free survival and overall survival in metastatic breast cancer patients [104]. The authors measured TNFα serum concentration from metastatic breast cancer patients receiving chemotherapy and found that a serum TNFα concentration of greater than 6.2 pg/mL correlated with a greater than 50% change of breast cancer survival [104].

Another group demonstrated that the use of infliximab, a monoclonal antibody that binds soluble and membrane bound TNFα, reduced outgrowth of osteolytic lesion formation in mice with bone metastatic breast cancer [105]. Hamaguchi et al. performed intracardiac injections of MDA-MB-231 human breast cancer cells, treated mice with or without infliximab, and monitored tumor formation for four weeks [105]. After four weeks, they found that mice treated with infliximab had decreased number of osteoclasts and decreased areas of osteolytic lesion formation [105]. In addition to infliximab, other anti-TNFα inhibitors, such as etanercept, have been used for to treat patients with metastatic breast cancer [106]. These publications, coupled with our results, demonstrate that TNFα is able to mediate osteoclast formation (Figure 8 and Figure 9). Thus, anti-TNFα therapies could be an interesting therapeutic for bone metastatic breast cancer patients.

We acknowledge several limitations to our study. First, osteoclastogenesis can occur via both RANKL dependent and RANKL independent mechanisms. For the study described here, we chose to focus on osteoclastogenesis via TNFα signaling, a RANKL independent mechanism, given that we found alterations in the expression of soluble TNFα over 50-fold in the conditioned media of EO cells compared to naïve osteoblasts (Figure 7a) compared to no change in the expression of soluble RANKL (Figure 7c). We acknowledge that other mechanisms may occur that regulate osteoclastogenesis in the presence of EO cells including the RANKL-LGR4 pathway, which we did not investigate [82]. Second, we chose to use a species-specific model of experimental metastasis (intratibial injections), which models established disease [53] for our in-vivo analysis. As a result, our system included human breast cancer cells and their effect on murine endogenous bone cells. While our in-vitro data, which consisted of mouse osteoclasts and mouse osteoblasts, well recapitulated what we observed in-vivo, we cannot rule out the possibility that there may be differences in the affinity of binding of factors that drive osteoclastogenesis between human and mouse cells in our in-vivo model. Third, we recognize that decreases in osteoclast DC-STAMP expression may be partly due to increased osteoclast apoptosis. Literature suggests that RAW 264.7 cells cultured with exogenous RANKL for extended periods of time experience periods of synchronized osteoclast differentiation and death [99]. While all pre-osteoclasts in our assays were only cultured for six days, we cannot rule out the possibility of apoptosis, which we did not directly assess. Fourth, while our data suggest that osteoclasts formed in the presence of EO cells or their conditioned media reduce osteoclast resorptive properties on a bone-like surface, and while we did estimate the resorptive ability of an individual osteoclast by calculation, we were unable to directly measure the resorptive ability of individual osteoclasts themselves. In addition, given that osteoclasts formed in the presence of EO cells or their conditioned media are less in number than osteoclasts formed in the presence of naïve osteoblasts, a reduction in amount of matrix resorbed may be due to reduced osteoclast number in the presence of EO cells.

**Figure 10:**
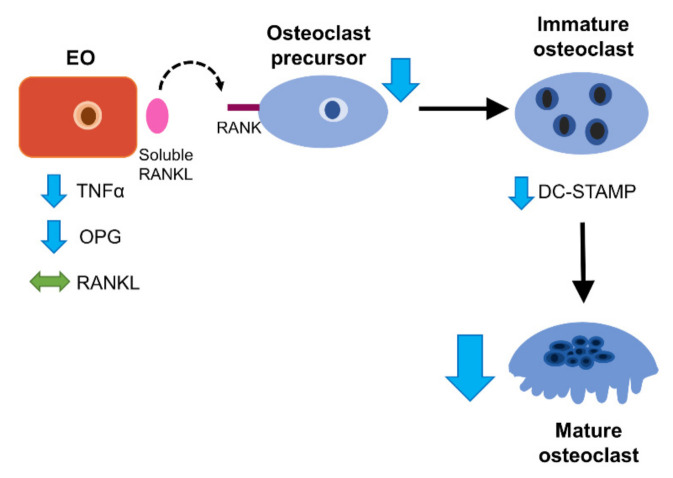
EO-Derived Factors Reduce Osteoclastogenesis. EO cells secrete soluble RANKL, which binds to the RANK receptor on osteoclast progenitor cells to initiate osteoclast differentiation. We found that EOs have decreased expression of osteoclastogenic factors OPG soluble protein and TNFα soluble protein. When determining the affect EO cells have on osteoclastogenesis, we found that osteoclasts exposed to EO-derived factors or produced in the presence of EO cells were less in number and smaller in size. When examining osteoclast fusion, we found that osteoclasts exposed to EO-derived factors have decreased expression of osteoclast-fusion protein DC-STAMP. Furthermore, osteoclasts produced in the presence of EO cells have decreased resorptive activity. Overall, the presence of EO cells decreases osteoclast formation and resorption, reducing osteoclastogenesis.

Our study herein aimed to identify how osteoclastogenesis is affected by EO cells. We showed that osteoclasts formed in the presence of EO cells or their conditioned medium are less in number, smaller in size, and resorb less bone matrix compared to osteoclasts produced in the presence of naïve osteoblasts (Figure 2, Figure 3 and Figure S2). We also demonstrated in-vivo that osteoclast formation was decreased in the presence of EO cells plus breast cancer cells (Figure 5). Patients with bone metastatic breast cancer usually present with osteolytic lesions, whereby osteoclasts are overactive and resorb excess bone to support cancer growth [107]. Osteoclasts drive breast cancer proliferation through the resorption of bone due to the release of sequestered cytokines and growth factors [35]. Importantly, our combined in-vitro and in-vivo data showed that osteoclast formation and resorption can be reduced by the presence of EO cells or EO-derived factors (Figure 10). Furthermore, our data suggest that increased presence of EO cells in the bone-tumor niche may slow down or reduce osteoclast formation. We further demonstrate that these effects may be modulated, in part, by TNFα from EO cells.

Lawson et al. used intravital imaging to track the dissemination of multiple myeloma cells to bone and determine the interactions between multiple myeloma cancer cells, osteoblasts, and osteoclasts in the endosteal niche [42]. Osteoblasts suppressed multiple myeloma cancer cell proliferation and maintained these cells in a dormant state during early-stage disease [42]. Conversely, the group demonstrated that exposure of multiple myeloma cells to osteoclasts in the niche promoted myeloma cell proliferation and reactivated them from their dormant state [42]. Our data has added an additional important piece to the puzzle, whereby we demonstrate that osteoclast formation in bone is also regulated by a subpopulation of osteoblasts, that is EO cells, and that osteoclastogenesis is reduced in the presence of the EO cells or their conditioned media. Importantly, we also found that this phenotype was maintained in the presence of bone metastatic breast cancer cells (Figure 4). Moreover, in murine tibia, we found that increased numbers of EO cells led to the production of osteoclasts that were smaller in size compared larger osteoclasts formed in bones containing increased numbers of naïve osteoblasts (Figure 5). Our data demonstrates that increased numbers of EO cells in the bone-tumor niche reduce osteoclastogenesis. 

During cancer progression in bone, factors produced by bone metastatic breast cancer cells recruit osteoblasts to promote osteoclast formation and increase bone degradation [65,108]. It is imperative to understand the interactions between breast cancer cells, osteoblasts, and osteoclasts and how these cell types work together to promote cancer progression. Our study aimed to identify the interaction between a novel subpopulation of osteoblasts, called EO cells, and their effect on osteoclast formation and resorption. Our data demonstrate that osteoclast formation and resorption is decreased in the presence of EO cells or their conditioned medium (Figure 2, Figure 3, and Figure S2). We also demonstrated in-vitro and in-vivo that osteoclasts formed in the presence of breast cancer cells and EO cells were less in number compared to osteoclasts produced in the presence of naïve osteoblasts (Figure 4 and Figure 5). We found that altered osteoclast formation by EO cells may be mediated in part by TNFα (Figure 8 and Figure 9). Our data demonstrate that osteoclastogenesis is reduced by EO cells, suggesting that EO cells have a protective effect in bone, and exert an inhibitory effect on tumor progression.

## 5. Conclusions

The focus of this study was to determine how osteoclastogenesis is influenced by EO cells. Osteoclasts are valuable as endogenous targets to aid in the prevention of excess bone degradation and are an important factor to determine disease status. Our data has shown that factors produced by EO cells reduce osteoclast formation and bone resorption. In breast cancer bone metastasis, osteoclasts become overactive and degrade bone at a faster rate than osteoblasts deposit new bone [107]. Our data in this publication demonstrate that osteoclasts produced in the presence of EO cells are less in number, smaller in size, and have decreased resorptive activity on a bone mimetic surface, which is, in part, mediated by TNFα.

## Data Availability

No new data were created or analyzed in this study. Data sharing is not applicable to this article.

## Supplementary Materials

The following are available online at https://www.mdpi.com/2072-6694/13/2/263/s1: Figure S1: Multinucleated, TRAP-positive Osteoclasts are Greater in Number Compared to Binucleated and Mononucleated TRAP-positive Osteoclasts, Figure S2: Primary Bone Marrow Monocytes Produced in the Presence of EO cells are Less in Number, Figure S3: Von Kossa Staining of Bone Mimetic Matrix, Figure S4: Multinucleated Osteoclasts are Found in the Bone-Tumor Microenvironment, Figure S5: EOs Decrease the Number of TRAP+, Multinucleated Osteoclasts in the Presence of GFP/luc2 MDA-MB-231 Human Breast Cancer Cells, Figure S6: Western blot analysis of DC-STAMP and β-actin on Day 3 samples, Figure S7: Western blot analysis of DC-STAMP and β-actin on Day 6 samples, and Figure S8: EO-mediated Osteoclast Formation using Primary Bone Marrow Monocytes can be Modulated by TNFα. Table S1: Estimation of Bone Resorptive Activity Per Individual Osteoclast.
