# Supplementary material for: ‘Educated’ Osteoblasts Reduce Osteoclastogenesis in a Bone-Tumor Mimetic Microenvironment
Source: Cancers (Basel). 2021 Jan 12;13(2):263. doi: 10.3390/cancers13020263 (PMC7828118; doi:10.3390/cancers13020263)
Supplement: Supplementary file 1 [file cancers-13-00263-s001.pdf]

cancers

MDPI

Supplementary Material: 'Educated' Osteoblasts Reduce Osteoclastogenesis in a Bone-Tumor Mimetic Microenvironment

Alexus D. Kolb, Jinlu Dai, Evan T. Keller and Karen M. Bussard

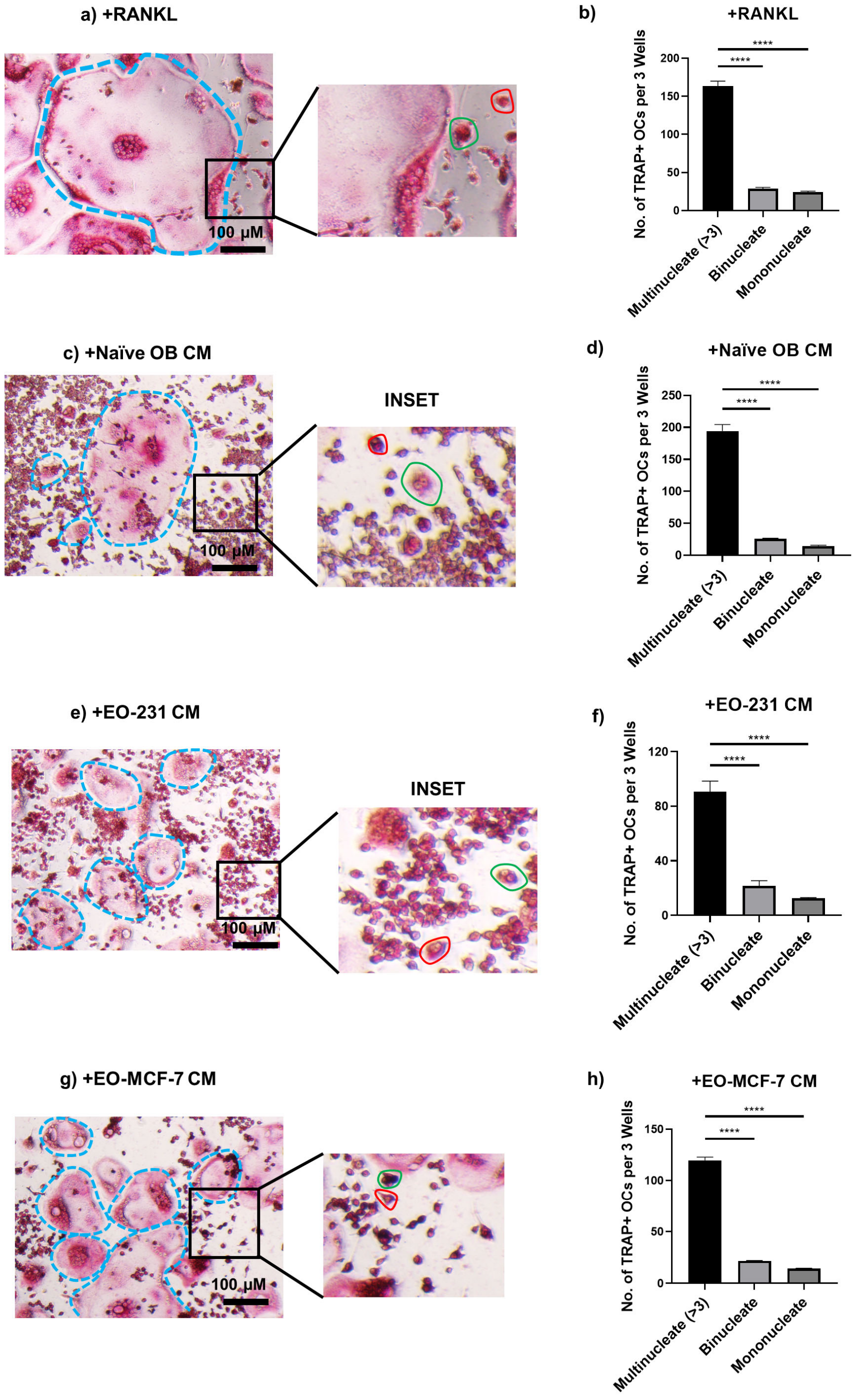

Figure 1. Multinucleated, TRAP-positive Osteoclasts are Greater in Number Compared to Binucleated and Mononucleated TRAP-positive Osteoclasts. RAW 264.7 pre-osteoclasts were cultured in the (**a**) presence of 50 ng/ml exogenous RANKL (control) and (**b**) the number (No.) of nuclei (multinucleated (≥3 nuclei), binucleated, and mononucleated) were quantified. Additionally, RAW 264.7 pre-osteoclasts were exposed to (**c**) naïve OB CM as an additional control and (**d**) the number (No.) of nuclei (multinucleated (≥3 nuclei), binucleated, and mononucleated) were quantified. For the first experimental condition, RAW 264.7 pre-osteoclasts were exposed to (**e**) EO-231 CM and (**f**) the number (No.) of nuclei (multinucleated (≥3 nuclei), binucleated, and mononucleated) were quantified. For the second experimental condition, RAW 264.7 pre-osteoclasts were exposed to (**g**) EO-MCF-7 CM and (**g**) the number (No.) of nuclei (multinucleated (≥3 nuclei), binucleated, and mononucleated) were quantified. All cultures were stained for tartrate-resistant acid phosphatase (TRAP; pink color), an established marker for mature osteoclasts. Blue, dashed outlines indicate TRAP-positive, multinucleated (≥3 nuclei) osteoclasts. Green outlines indicate TRAP-positive, bi-nucleated osteoclasts. Red outlines indicate TRAP-positive, mononucleated osteoclasts. Black boxes indicate inset location. Scale = 100 μM. $N = 3$. ***** $p < 0.0001$.

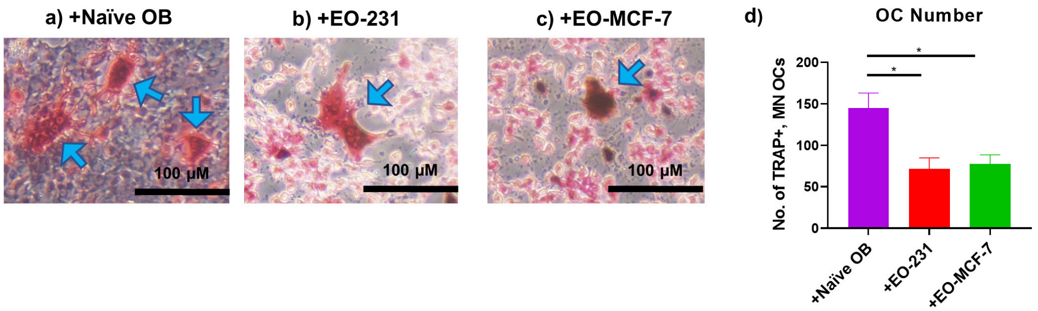

Figure S2. Primary Bone Marrow Monocytes Produced in the Presence of EO cells are Less in Number. CD11b+ primary bone marrow monocytes (BMMs) were collected as described in the Materials and Methods section. CD11b+ BMMs were co-cultured with (**a**) naïve OBs (control), (**b**) EO-231 cells, or (**c**) EO-MCF-7 cells in the presence of exogenous M-CSF and exogenous RANKL for 6 days. After 6 days, cultures were stained for tartrate-resistant acid phosphatase (TRAP; pink color), an established marker for mature osteoclasts. Blue arrows depict TRAP-positive, multinucleated (≥3 nuclei) osteoclasts. The number (No.) of TRAP-positive, multinucleated (≥3 nuclei) osteoclasts were quantified for each condition (**d**). Scale = 100 μM. $N = 3$. * $p < 0.05$.

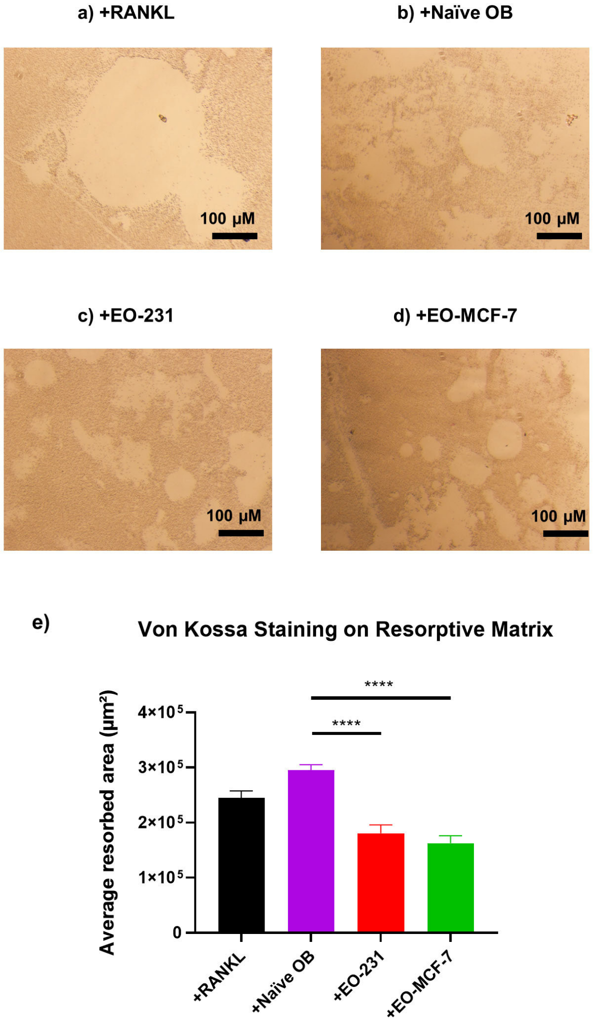

Figure S3. Modified Von Kossa Staining of Bone Mimetic Matrix. RAW 264.7 pre-osteoclasts were co-cultured on a bone mimetic matrix in the presence of 50 ng/ml exogenous RANKL (**a**) and co-cultured with either (**b**) naïve OBs (control), (**c**) EO-231 cells, or (**d**) EO-MCF-7 cells in the presence of 50 ng/ml exogenous RANKL for 6 days. A modified von Kossa staining was used to visualize resorptive pits. The average resorbed area was quantified for each condition (**e**). At least three individual batches were assayed per condition. Resorptive pits were visualized via light microscopy. Scale = 100 μM. $N = 3$. **** $p < 0.0001$.

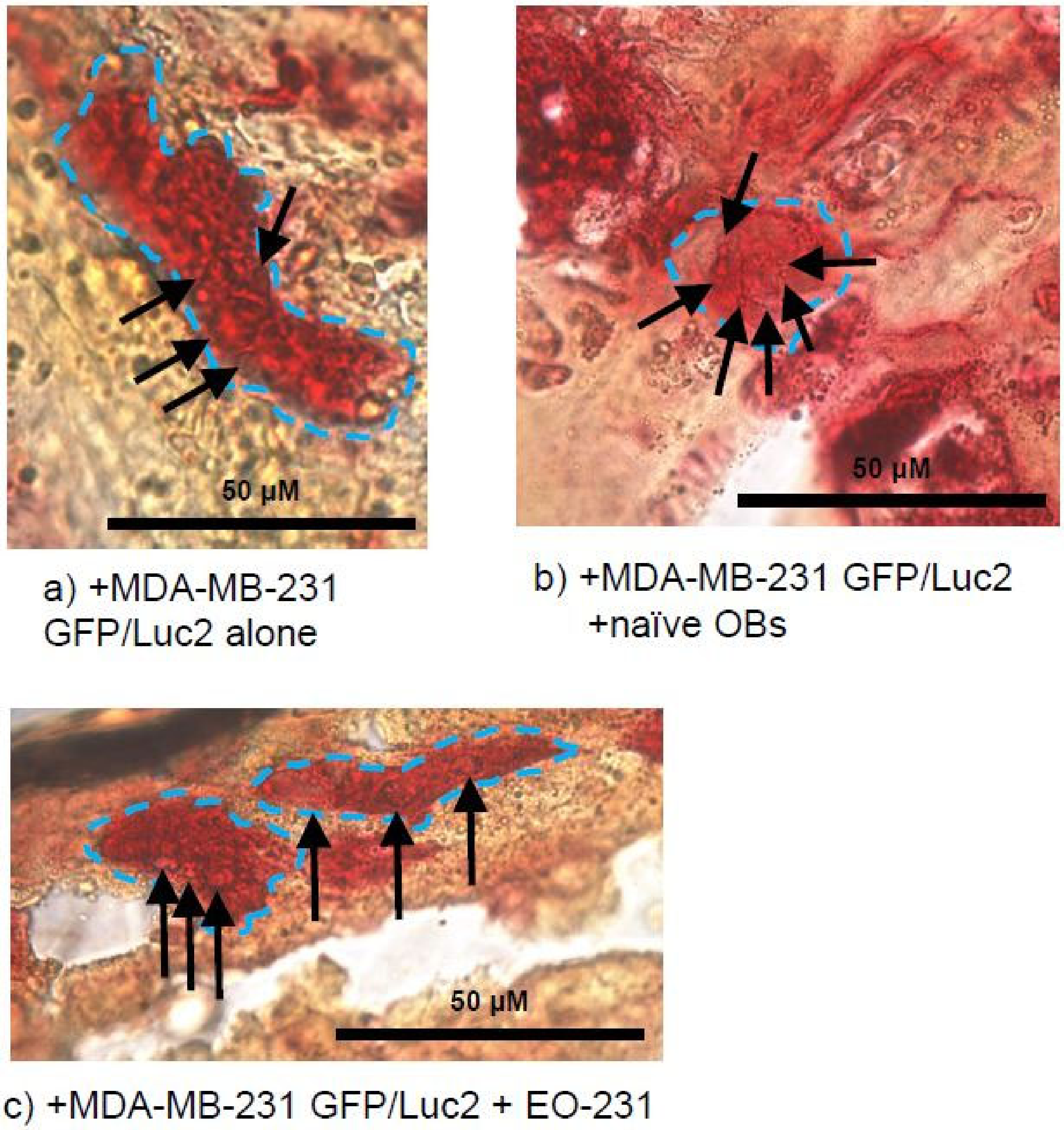

Figure S4. Multinucleated Osteoclasts are Found in the Bone-Tumor Microenvironment. Athymic nude mice were injected via intratibial injection with (**a**) MDA-MB-231GFP/Luc2 human breast cancer cells alone or (**b**) an admix of MDA-MB-231GFP/Luc2 human breast cancer cells plus naïve osteoblasts, or (**c**) an admix of MDA-MB-231GFP/Luc2 human breast cancer cells plus EO-231 cells. Eight weeks later, mice were euthanized and their tibia harvested. Tibia sections were stained for tartrate-resistant acid phosphatase (TRAP; blue dashed outlines), a common marker for mature osteoclasts. One representative image is displayed per condition. Black arrows indicate nuclei of TRAP-positive, multinucleated (≥3 nuclei) osteoclasts. Scale = 50µM.

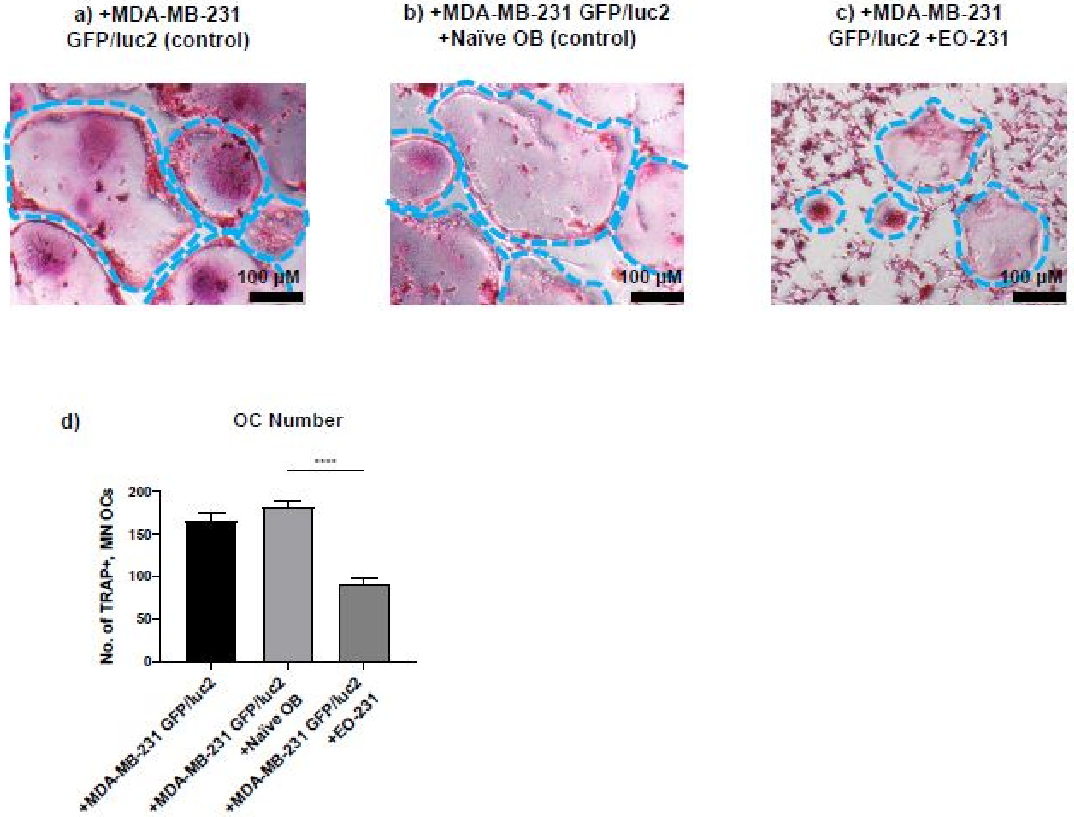

Figure S5. EOs Decrease the Number of TRAP-Positive, Multinucleated Osteoclasts in the Presence of GFP/luc2 MDA-MB-231 Human Breast Cancer Cells. RAW 264.7 pre-osteoclasts were cultured with (**a**) MDA-MB-231GFP/luc2 human breast cancer cells in the presence of 50 ng/ml exogenous RANKL alone for 6 days (control). RAW264.7 pre-osteoclasts were co-cultured with either (**b**) MDA-MB-231GFP/luc2 human breast cancer cells plus naïve OBs in the presence of 50 ng/ml exogenous RANKL (control) or (**c**) MDA-MB-231GFP/luc2 human breast cancer cells plus EO-231 cells in the presence of 50 ng/ml exogenous RANKL for 6 days. After 6 days, all conditions were stained for TRAP (blue outlines), a common marker for mature osteoclasts. The number (No.) of TRAP-positive, multinucleated (≥3 nuclei) mature osteoclasts were quantified for each condition (**d**). One representative image is shown per condition. Scale = 100 µM. *N*=3. **** $p < 0.0001$.

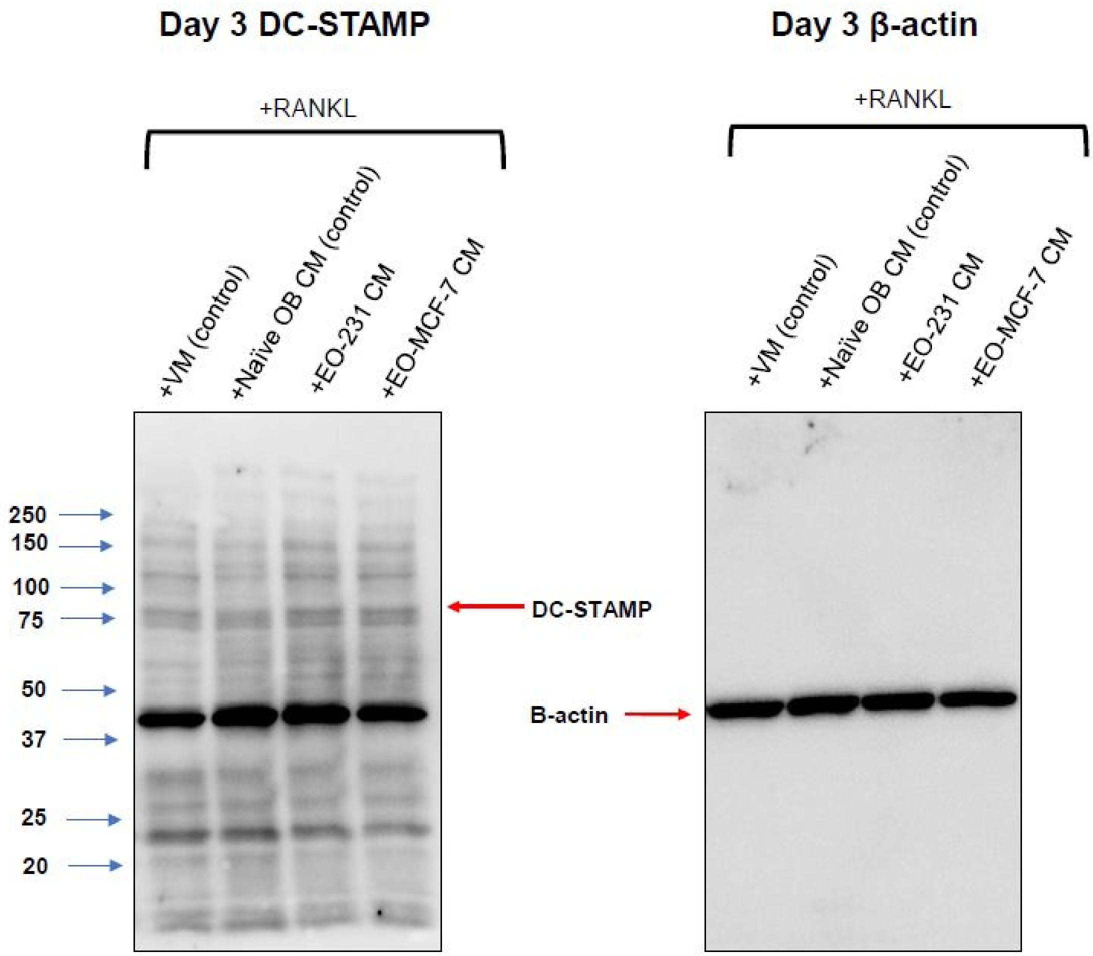

Figure 6. Western blot analysis of DC-STAMP and β-actin on Day 3 samples. Uncropped image used for Figure 6A. Samples order: Day 3 differentiated RAW 264.7 pre-OCs in vehicle media (VM); Day 3 differentiated RAW 264.7 pre-OCs exposed to naïve OB CM; Day 3 differentiated RAW 264.7 pre-OCs exposed to EO-231 CM; Day 3 differentiated RAW 264.7 pre-OCs exposed to EO-MCF-7CM.

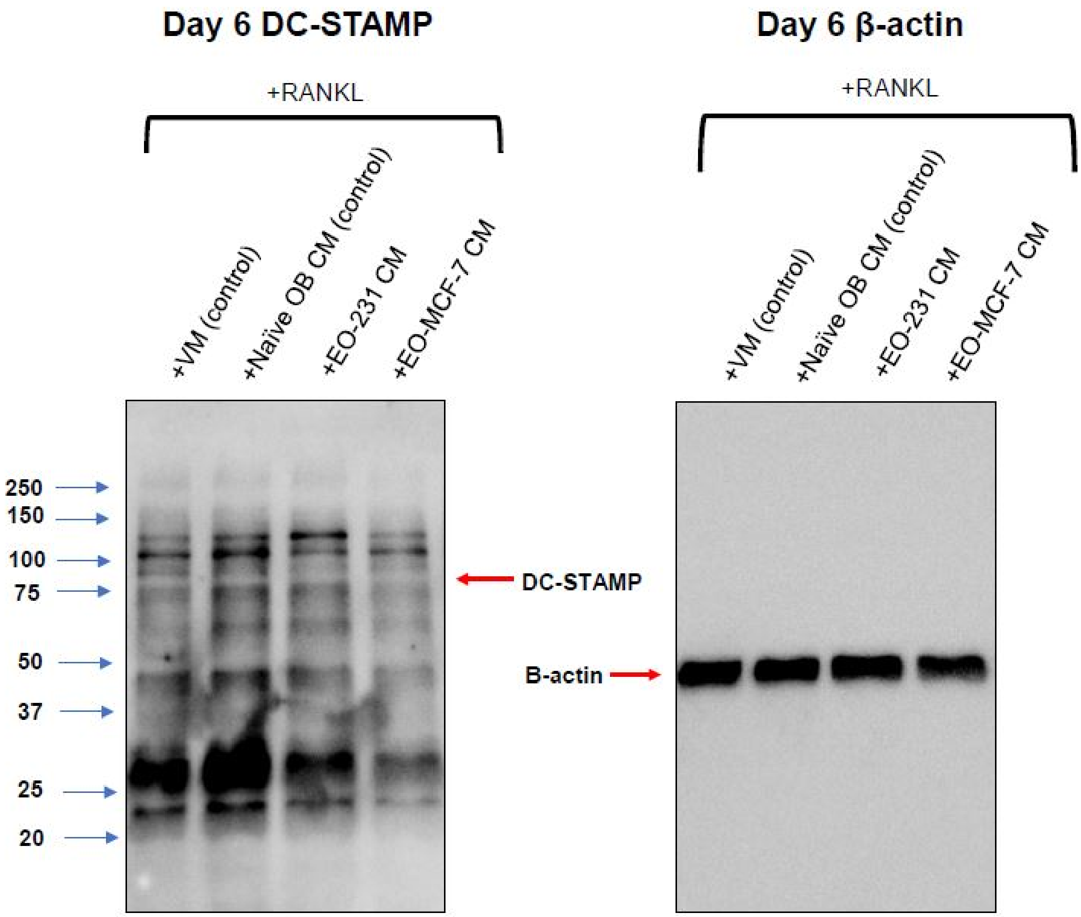

Figure S7. Western blot analysis of DC-STAMP and β-actin on Day 6 samples. Uncropped image used for Figure 6B. Samples order: Day 6 differentiated RAW 264.7 pre-OCs in vehicle media (VM); Day 6 differentiated RAW 264.7 pre-OCs exposed to naïve OB CM; Day 6 differentiated RAW 264.7 pre-OCs exposed to EO-231 CM; Day 6 differentiated RAW 264.7 pre-OCs exposed to EO-MCF-7 CM.

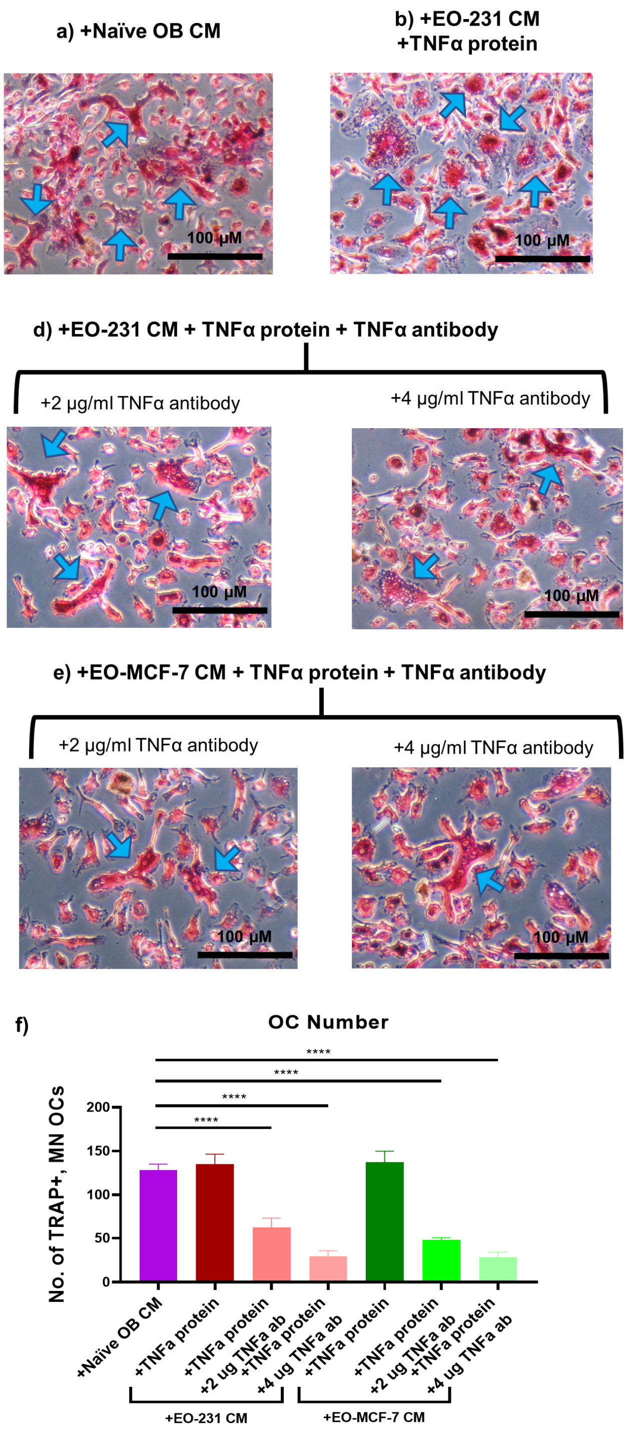

Figure S8. EO-mediated Osteoclast Formation using Primary Bone Marrow Monocytes can be Modulated by TNFα. CD11b+ primary bone marrow monocytes (BMMs) were collected as described in the Materials and Methods section. CD11b+ BMMs were exposed to (**a**) naïve OB CM (control), (**b**) EO-231 CM plus TNFα recombinant protein (12 ng/ml), (**c**) EO-MCF-7 CM plus Table 12. ng/ml), (**d**) EO-231 CM plus TNFα recombinant protein (12 ng/ml) plus TNFα neutralizing antibody (2 μg/ml or 4 μg/ml), or (**e**) EO-MCF-7CM plus TNFα recombinant protein (12 ng/ml) plus TNFα neutralizing antibody (2 μg/ml or 4 μg/ml). All cultures were maintained in the presence of exogenous M-CSF and exogenous RANKL for 6 days. After 6 days, cultures were stained for tartrate-resistant acid phosphatase (TRAP; pink color), an established marker for mature osteoclasts. Blue arrows depict TRAP-positive, multinucleated (≥3 nuclei) osteoclasts. The number (No.)

of TRAP-positive, multinucleated (≥3 nuclei) osteoclasts were quantified for each condition (**f**). Scale = 100 μM. $N = 3$. **** $p < 0.0001$.

Table 1. Estimation of Bone Resorptive Activity Per Individual Osteoclast.

Condition	Avg. Resorbed Area (μm^2)*	Avg. No. of TRAP-positive, multinucleated OCs#	Estimated Resorbed Activity by an Individual Osteoclast†
+RANKL	2706.29	173.75	15.57577
+Naïve OB CM	2452.91	312.2	7.856855
+EO-231 CM	1248.31	251.857	4.956424
+EO-MCF-7 CM	1038.25	192.429	5.395497

*avg resorbed area (μm^2) values for each condition as per Figure 3f. # avg number (No.) of TRAP-positive, multinucleated OCs values for each condition as per Figure 3e. †avg resorbed area/avg No. TRAP-positive, multinucleated OCs = estimated resorbed activity by an individual osteoclast RAW 264.7 pre-osteoclasts were co-cultured on a bone mimetic matrix with either naïve OBs (control), EO-231, or EO-MCF-7 in the presence of 50 ng/ml RANKL for 6 days. RAW 264.7 pre-osteoclasts on a mimetic bone matrix in the presence of 50 ng/ml RANKL for 6 days served as a control. The estimated resorptive activity of an individual osteoclast was quantified by dividing the average resorbed area by the average number of TRAP-positive, multinucleated osteoclasts (OCs) for each condition. The values for average resorbed area and the average number of osteoclasts for each condition were used from Figure 3f and Figure 3e, respectively.
